# Maskless, rapid manufacturing of glass microfluidic devices using a picosecond pulsed laser

**DOI:** 10.1038/s41598-019-56711-5

**Published:** 2019-12-27

**Authors:** Krystian L. Wlodarczyk, Duncan P. Hand, M. Mercedes Maroto-Valer

**Affiliations:** 1 Research Centre for Carbon Solutions (RCCS), Institute of Mechanical, Process and Energy Engineering, School of Engineering and Physical Sciences, Heriot-Watt University, Edinburgh, United Kingdom; 2 Institute of Photonics and Quantum Sciences, School of Engineering and Physical Sciences, Heriot-Watt University, Edinburgh, United Kingdom

**Keywords:** Biogeochemistry, Environmental sciences, Hydrology, Engineering, Optics and photonics, Physics

## Abstract

Conventional manufacturing of glass microfluidic devices is a complex, multi-step process that involves a combination of different fabrication techniques, typically photolithography, chemical/dry etching and thermal/anodic bonding. As a result, the process is time-consuming and expensive, in particular when developing microfluidic prototypes or even manufacturing them in low quantity. This report describes a fabrication technique in which a picosecond pulsed laser system is the only tool required to manufacture a microfluidic device from transparent glass substrates. The laser system is used for the generation of microfluidic patterns directly on glass, the drilling of inlet/outlet ports in glass covers, and the bonding of two glass plates together in order to enclose the laser-generated patterns from the top. This method enables the manufacturing of a fully-functional microfluidic device in a few hours, without using any projection masks, dangerous chemicals, and additional expensive tools, e.g., a mask writer or bonding machine. The method allows the fabrication of various types of microfluidic devices, e.g., Hele-Shaw cells and microfluidics comprising complex patterns resembling up-scaled cross-sections of realistic rock samples, suitable for the investigation of CO_2_ storage, water remediation and hydrocarbon recovery processes. The method also provides a route for embedding small 3D objects inside these devices.

## Introduction

Microfluidic devices are tools that allow the analysis, manipulation and processing of tiny amounts of fluids, often smaller than a droplet of water (<10 μL)^1^. The devices are typically made of two plates, one of which has a microfluidic pattern generated on its surface, whereas the other has a set of inlet/outlet ports and is used as a lid to enclose the microfluidic pattern from the top. Typically, the lid is transparent and must be properly bonded to the other plate such that the device is leak-proof and the injected fluids can flow only within the area of a microfluidic pattern. Such microfluidic devices enable various complex physical and chemical operations to be performed on small amounts of liquids, gases and solids, including small particles, colloids, living cells and microbes. Therefore, these devices have found use in many different areas of chemistry, biology and medicine^1–5^. So-called Micro Total Analysis Systems (μTAS), Lab-on-a-chip (LoC) devices, Organ-on-a-Chip (OoC) devices, and Point-of-Care (PoC) medical diagnostic devices are used by biochemists, pharmacologists, clinicians and medical staff. These devices, for instance, allow them to analyse and manipulate single cells^6–8^, detect pathogens and diseases^9–12^, discover and develop new drugs and antibiotics^13,14^, or even mimic human and animal organs^15–17^. Microfluidic devices also play an important role in research related to hydrology, carbon dioxide storage and hydrocarbon recovery^18–25^. In these fields of science, they are used as simplified models and up-scaled replicas of porous media (e.g. rock samples) for the investigation of different fluid flow phenomena that govern surface and subsurface systems. Thanks to these devices, it is possible to observe and study different multiphase fluid flow mechanisms that occur for instance between rock grains, such as snap-off and corner flow phenomena which are responsible for disconnection and trapping of carbon dioxide and/or hydrocarbons^26^.

Microfluidic devices can be manufactured from a wide range of materials, such as transparent silicone elastomer (poly-di-methyl-siloxane (PDMS)), thermoplastics (e.g. poly-methyl-methacrylate (PMMA), polycarbonate (PC), cyclic-olefin-copolymer (COC)), renewable polymers (e.g. polylactic acid (PLA)), chromatography paper, photoresist, hydrogels, glass and silicon^26–35^. Depending on the material used, a microfluidic pattern can be generated by using soft lithography, casting, hot embossing, injection moulding, wax printing, inkjet printing, mechanical milling, laser micro-machining, two-photon polymerisation, etching or even 3D printing^36–42^.

Glass in comparison to the other materials used offers a unique combination of optical, mechanical, thermal, electrical and chemical properties. Superior optical transparency, high hardness, thermal stability and temperature resistance, excellent electrical isolation, chemical inertness to many fluids, biocompatibility, and well-defined surface chemistry make this material almost ideal for the fabrication of microfluidic devices, in particular those for the investigation of CO_2_ sequestration and hydrocarbon recovery processes where different (often aggressive, reactive and flammable) fluids can be injected at very high pressures (even > 300 bar) and elevated temperatures. Unfortunately, conventional manufacturing of glass microfluidic devices is a complex, multi-step process that involves using a combination of different fabrication techniques and tools^36–38^. As a result, it is time-consuming and expensive in particular for the manufacture of laboratory microfluidics in low quantity. The most common techniques used for the generation of microfluidic patterns on glass substrates are reactive ion etching (RIE) and wet (chemical) etching, which respectively use chemically reactive plasma and liquid chemicals called etchants to remove selectively the material. Both these methods require the use of custom-designed masks that certainly adds time, cost and efforts to generate a pattern. These masks can be manufactured by using a combination of spin coating and direct laser writing, physical vapour deposition and direct laser writing, photolithography and etching, or photolithography and electroplating. Microfluidic patterns can also be generated directly on a glass surface using a CO_2_ or ultrashort pulsed laser^43–47^, or inside the material using so-called a selective laser etching (SLE) process^48–50^. The latter method is particularly attractive because it eliminates additional fabrication steps related to the bonding of two glass plates together, but it still requires the use of etchants.

To ensure the flow of fluids only within the area of a microfluidic pattern, the pattern must be properly closed from the top using the second glass plate. Two glass plates can be permanently bonded together using adhesives, but this method has a high risk that glue will enter microchannels during the bonding process, blocking and destroying the flow pattern. More common methods involve the use of intermediate layers (such as SU-8 photoresist, parylene, polyimide), heat (so-called thermal bonding) or electric field (so-called anodic bonding)^36,37,47^. Unfortunately, all these methods require additional equipment, e.g. a furnace, wafer bonding machine and clean room, which again significantly increases the fabrication costs of glass microfluidic devices.

**Figure 1:**
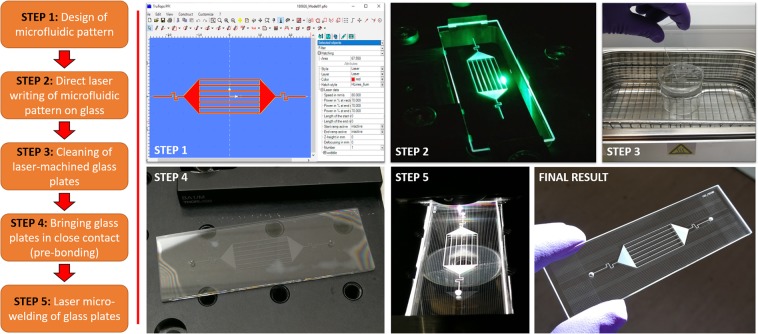
Key stages of the laser-based technique for the rapid fabrication of glass microfluidic devices. STEP 1 involves the design of a microfluidic pattern and importing it to the laser machining software (Trumpf Trutops PFO). In STEP 2, the laser generates the microfluidic pattern directly on the glass surface by ablating the material. In this step, inlet/outlet ports are also generated in the second glass plate. In STEP 3, the glass plates are cleaned in order to remove debris and burrs generated during the laser machining process. STEP 4 involves bringing the laser-machined glass plates into optical contact. This process is called pre-bonding. STEP 5 is a laser microwelding process in which the two glass plates are permanently bonded together without using any adhesives nor intermediate layers. The welding closes any pre-existing gaps between the two materials, making the device leak-proof and free of air gaps. All photographs were taken during different fabrication stages of a microfluidic device.

This report describes a maskless laser-based method suitable for the fabrication of glass microfluidic devices (see Fig. 1), where an ultrashort picosecond pulsed laser system is the only tool used for the manufacture of the entire devices. In contrast to the other fabrication methods described earlier, this technique does not use any dangerous etchants (e.g. hydrofluoric acid) and additional expensive equipment, and also it can be carried out in normal laboratory environment (i.e. without being in a cleanroom).

The concept and initial results of our fabrication method were presented in the Micromachines journal^51^. Since then, we have improved significantly the fabrication process. For instance, we found a solution to eliminate submicron gaps between the two glass substrates and developed a technique for the manufacture of microfluidic devices using thinner (≤0.5 mm) glass substrates. As will be shown in this report, our fabrication process enables the rapid manufacturing of different types of microfluidics, e.g., Hele-Shaw cells^52^ or microfluidic devices comprising patterns that resemble up-scaled cross-sections of realistic rock samples. Such devices are used in many areas of science and engineering (CO_2_ sequestration, water remediation, petroleum engineering) as micromodels of porous media for the investigation of different multiphase fluid flow phenomena occurring in surface and subsurface systems. Our fabrication method can also be adapted to encapsulate small 3D objects inside microfluidic patterns. This unique feature of the process opens an opportunity to manufacture glass microfluidic devices with integrated minerals and/or miniature sensors, which can be used for monitoring different dynamic parameters (e.g. pressure, temperature or pH change) inside channels as fluid flows are introduced.

## Materials and Methods

### Glass

75 mm × 25 mm borosilicate glass plates (Borofloat 33, SCHOTT Technical Glass Solutions GmbH, Germany) of nominal thickness 1.1 mm were used to manufacture the microfluidic devices presented in this report; thinner plates (0.25 mm and 0.5 mm thick) were also used for the Hele-Shaw cells. Borofloat 33 glass was chosen because this material has many physical properties similar to highly pure fused silica, but is less expensive. It is characterised by very high transparency, outstanding thermal resistance, high chemical durability and excellent mechanical strength^53^. The glass plates were purchased from Newcastle Optical Engineering Ltd. (UK).

### Laser

The direct writing of microfluidic patterns, the drilling of inlet/outlet ports, and the bonding of the glass plates was performed using a picosecond pulsed laser (TruMicro 5x50, Trumpf GmbH, Germany). The maximum average power (P_MAX_) of this laser is 50 W, as measured at the fundamental wavelength (λ) of 1030 nm and the pulse repetition frequency (PRF) of 400 kHz, the maximum pulse energy (E_PMAX_) is 125 μJ, and the pulse duration is 6 ps, as measured at Full-Width-Half-Maximum. The laser is switchable between three wavelengths: 1030 nm, 515 nm and 343 nm. Each output provides a linearly polarised laser beam. The P_MAX_ values for the shorter wavelengths (515 nm and 343 nm) are 32 W and 16 W, respectively.

### Laser micromachining setup

**Figure 2:**
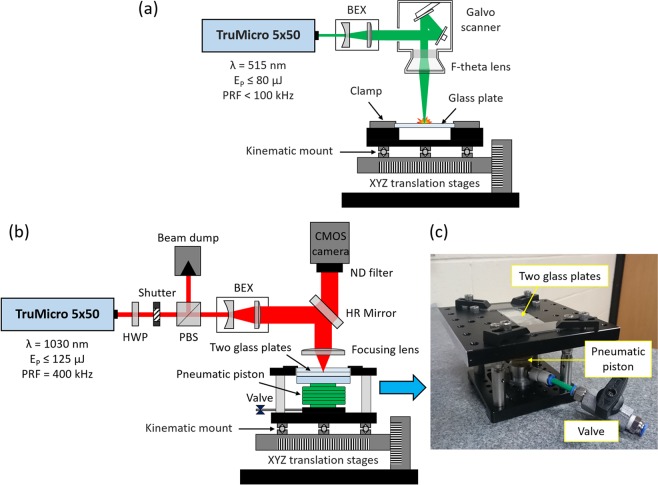
Schematics of laser setups used for: (**a**) micromachining and (**b**) microwelding of glass plates, and (**c**) a photograph of the custom holder used for welding the glass plates. Acronyms: BEX – beam expander; HWP – half-wave plate; PBS – polarising beam splitter; HR mirror – high reflectivity mirror with polished back surface; ND filter – neutral density filter.

Microfluidic patterns were generated using the laser setup shown schematically in Fig. 2(a). The laser micromachining of glass was carried out using the 515 nm wavelength. The laser beam radius in the focus (ω_0_) was approximately 12 μm, as measured at 1/e^2^ of its peak intensity, using a scanning slit beam profiler (Beam-Map 2, DataRay, US). Such a small laser spot was obtained by expanding the output laser beam to a 6.4 mm diameter spot and then focusing it using a 163 mm focal length F-Theta lens mounted to a galvo scanner (HSR10, Trumpf, Germany). The laser was controlled using TruControl 1000 software (Trumpf, Germany). The galvo scanner enables movement of the laser beam with a velocity as high as 5 m/s. The glass plates ready for machining were placed in a customised holder, which later was mounted onto the XYZ linear translation stages (PRO115-05MM, Aerotech Inc., US). A kinematic mount attached to the holder and the base of the translation stages allowed us to obtain repeatable positioning of the glass plates for micromachining.

### Laser microwelding setup

The bonding of the laser-machined glass plates was performed using the setup shown schematically in Fig. 2(b). In this setup, the 1030 nm beam is delivered to the glass plates through a half-wave plate (HWP), polarising beam splitter (PBS), beam expander (BEX) and 10 mm focal length aspheric lens (AL1210M-B, Thorlabs, US). The HWP and PBS are used to reduce the laser beam power in a controlled way, allowing an appropriate power level to be selected. The high-reflectivity (HR) mirror between the BEX and the focusing lens has a polished back surface that partially transmits the laser beam reflected from the top surface of the upper glass plate to a 10 megapixel CMOS camera (uEye UI-1490RE, IDS Imaging Development Systems GmbH, Germany). The camera does not have any in-built focusing optics, only a neutral density (ND) filter is attached for attenuating the laser beam. This allows the Fresnel reflection from the air-glass interface to be imaged onto the CMOS sensor by using the 10 mm FL aspheric lens. By adjusting the lens-camera separation distance, it is possible to obtain this image and determine the focal position of the laser beam relative to the glass plate surface. The glass plates prepared for welding are mounted in a special holder, which is shown in Fig. 2(c), and placed onto the XYZ linear translation stages. The holder consists of a pneumatic piston that applies an upwards force to the lower glass plate, ensuring that close contact between the two plates is maintained throughout the welding process. Similarly to the laser micromachining setup, the holder has a kinematic mount which provides repeatable positioning of the glass plates for microwelding.

### Laser micromachining procedure

Microfluidic flow patterns and inlets/outlets ports were generated using the 515 nm laser wavelength. At this wavelength, the laser provides the highest peak fluence and enables the generation of features as small as 20 μm. The laser uses TruTops PFO software that enables the generation of various microfluidic patterns by raster scanning the laser beam. This scanning method was described in our other publication^54^. In TruTops PFO software the laser machining parameters, such as pulse energy (E_P_), laser beam scan speed (v) and the number of laser passes (N), can be defined for each line and polyline. For the generation of more complex patterns, we used AutoCAD software where the final design was saved as a DXF file and exported to TruTops PFO. Laser machining of glass plates was performed in ambient air without using any additional gasses. Inlet/outlet ports in a borosilicate glass cover were generated by machining multiple times the same area of glass until through holes were created.

### Cleaning the laser-machined glass plates

Following the laser machining process, each glass plate was placed individually into a beaker filled with methanol. Then the beaker was placed into an ultrasonic cleaner (USC-300-THD, VWR International, UK) for a few minutes at room temperature in order to remove loose debris from the glass surfaces. In addition to loose debris, the laser machining process generates small burrs around the machined area, which cannot be removed in this way, hence the glass surfaces were also gently lapped by hand. For lapping, we used a 0.3 μm FibrMet abrasive disc (Buehler, Germany) mounted onto a 10 mm thick glass plate. The cleanliness of the glass plates was checked using a Leica optical microscope (DM600 M, Leica, UK).

### Pre-bonding

Close contact between the glass cover and the glass plate comprising a microfluidic pattern must be obtained in order to achieve good welding results, i.e., high bonding strength, no material cracking, no ablation and no air gap between the two materials. Good contact is necessary at least in the area where the laser welding process starts. To obtain good contact, both glass plates were again washed in methanol, wiped off using soft polyester tissues (WW-3009, CRTM CleanRoomProducts GmbH, Germany), and treated with a jet of ionised nitrogen (Model 3080 S, Hugle Electronics Inc., Japan). Following this cleaning procedure, the glass cover was placed on the top of the other glass plate and pressed by hand. Perfect (optical) contact is achieved when optical fringes between the two materials disappear. If any large dust particles are trapped between the two glass plates, optical contact cannot be achieved and the cleaning process must be repeated; in this case it is necessary to first use the ultrasonic cleaner to separate the pre-bonded glass plates.

### Laser microwelding

Pre-bonded glass plates were placed in a special holder (see Fig. 2(c)), aligned in a slot, and pressed from the bottom by the pneumatic piston, which was pressurised with nitrogen to around 0.8 bar. This holder was transferred onto the base of the translation stages. Thanks to the kinematic mount that ensures repeatable positioning of the glass plates, it is possible to predict the position of the weld that will be generated by the laser beam for the given XYZ coordinates. This feature is very important because it allows us to control the generation of welds in specific areas, thereby avoiding the generation of welds inside the microfluidic channels.

Welds were generated along the glass-glass interface by opening a mechanical shutter (SH05/M, Thorlabs, Germany) and simultaneously moving the translation stages. The shutter and translation stages were controlled using a G-Code programming language in Ensemble Motion Composer (Aerotech Inc., US). The Aerotech software enables the creation of G-code scripts for the automatic generation of weld seams in specific areas in glass, for example, only around microfluidic patterns. In order to bond two glass plates together, the laser beam was focused below the glass-glass interface. After finding the Fresnel reflection from the first air-glass interface, the Z axis (vertical) stage was translated by an amount calculated based on the glass thickness and refractive index to a new position at which the laser beam was focused inside the material.

### Sample analysis

The mean depth and surface roughness of the laser-machined areas were measured using a non-contact surface profilometer (InfiniteFocus, Alicona Imaging GmbH, Austria). The measurements were performed using a × 10 objective that provides a field of view of 1.43 mm by 1.08 mm and a lateral resolution of 0.88 μm. The mean depth and surface roughness were calculated for areas of 0.5 mm by 0.5 mm.

The Leica optical microscope was also used to analyse the weld seams generated inside cross-sectioned glass samples. The cross-sectioning was performed by using a low-speed diamond wheel saw (STB Model 650, South Bay Technology, US). Small pieces of the sectioned glass were mounted in epoxy resin (Acri-Kleer, MetPrep, UK), and then mechanically polished using a lapping machine, (LaboPol-5, Struers, Germany) with a 3 micron diamond suspension (MetPrep, UK).

### Testing the microfluidic devices

Two different tests were performed. The first test was to determine whether the manufactured microfluidic device is watertight, whilst the second was to investigate fluid propagation through the microfluidic channels and determine whether the as-machined surface finish on the channel bottom and side-walls has any impact on flow. In the first experiment, we injected deionised (DI) water into the microfluidic device (to fill in the pattern completely), and then performed drainage of the device by injecting nitrogen. Different flow rates were used in order to validate the strength of welds. During the drainage process, we monitored the injection pressure using an analogue pressure gauge (available from RS Components, UK). Following each test, the device was visually inspected for any water leakage. In the second test, we delivered a small droplet of DI water to one of the ports by using a syringe needle. The propagation of water inside the patterns was observed and recorded using the Leica optical microscope.

## Results and Discussion

### Laser-generated microfluidic patterns

The mean depth (D) and surface roughness (S_a_) of the patterns generated using the 515 nm wavelength were observed to depend on many laser machining parameters, such as the laser spot size (2ω), pulse energy (E_P_), pulse repetition frequency (PRF), scan velocity (v), hatch distance (ΔH), and number of laser passes. Also the laser beam scanning strategy may have a significant impact on these two surface parameters (D and S_a_), as recently reported in Micromachines^54^. In general, too high PRF value, too low v, or too small ΔH may result in the generation of highly irregular surfaces that can contain thin glass fibres and glass particles partially fused to the surface. Obviously, such surfaces must be avoided in microfluidic patterns because they can introduce artefacts during the flow of fluids, making the system behaviour difficult to predict and model.

**Figure 3:**
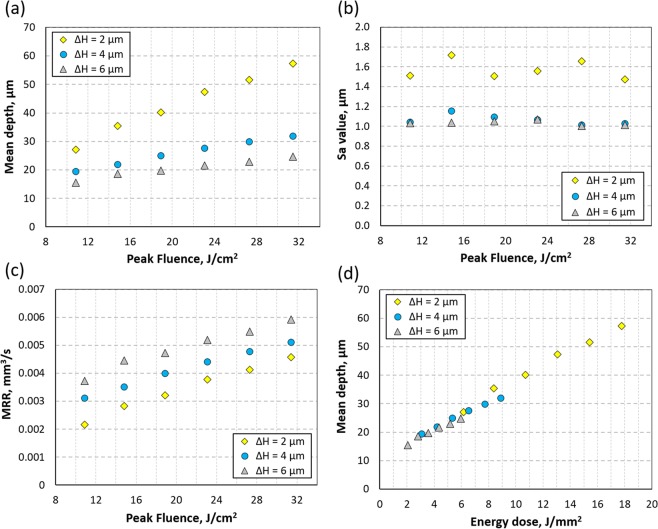
Laser micro-machining of Borofloat 33 glass. Influence of peak laser fluence on: (**a**) mean depth, (**b**) surface roughness, and (**c**) material removal rate (MRR) for different values of hatch distance (ΔH = 2, 4, and 6 μm). Also it was found that the mean depth increases linearly with increasing energy dose, as shown in (**d**). All data are valid for λ = 515 nm, 2ω_0_ = 24 μm, PRF = 20 kHz, v = 40 mm/s. Datasets used to plot these graphs are in the Supplementary Materials (see Table S1).

The microfluidic patterns presented in this report were generated using 2ω = 24 μm, PRF = 20 kHz, and v = 40 mm/s. By changing the peak fluence (F) and ΔH, as shown in Fig. 3(a), it was possible to generate patterns with different depths, in the range of 15 to 58 μm in a single laser pass. The peak fluence is defined as (2 × E_P_) / (π × ω^2^), where ω is the laser beam radius. To make deeper structures, multiple laser passes can be used (see Table S1 in the Supplementary Materials). Unfortunately, structures less than 15 μm deep cannot be generated because a laser peak fluence below 10.5 J/cm^2^ leads to partial machining of both glass surfaces (upper and lower) as a result of the incubation of low-intensity laser pulses and self-focusing of the laser beam in the transparent material^55^. The roughness (S_a_) of the laser-machined surfaces was measured to be approximately 1 μm for ΔH = 4 μm and 6 μm and approximately 1.6 μm for ΔH = 2 μm. As shown in Fig. 3(b), the S_a_ value varies slightly with peak fluence and this variation was observed to be greater for ΔH = 2 μm.

Material removal rate (MRR) is also an important parameter to consider as it directly impacts fabrication time, and this is plotted as a function of peak fluence in Fig. 3(c) for 3 different values of ΔH. Not surprisingly, a higher laser fluence results in an increase of MRR. More interestingly, however, for the same laser fluence an increase of ΔH from 2 μm to 6 μm increases the MRR by at least 25%. Finally, it was observed by calculating the energy dose (E_DOSE_) per 1 mm^2^ (calculated as N × E_P_, where N is the total number of laser pulses within a 1 mm^2^ area) that the mean depth of the laser-machined areas is a linear function of this parameter (see Fig. 3(d)). From a practical point of view, this means that microfluidic patterns of a certain depth can be generated by using different combinations of laser machining parameters, for instance, using a reduced laser fluence and a shorter hatch distance. Moreover, this also means that the same machining results can be obtained using a different picosecond laser system as long as the same energy dose and laser spot size are preserved.

### Cleaning and lapping the laser-machined glass plates

**Figure 4:**
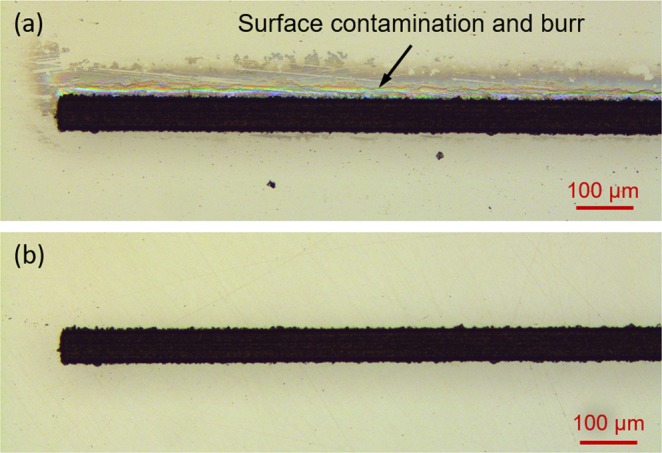
Laser-machined glass plate: (**a**) before and (**b**) after the cleaning and lapping.

The laser-machined patterns are typically surrounded by glass particles and have small (sub-micron in height) but well-attached burrs. These defects must be removed prior to the bonding process in order to obtain close contact and eliminate the formation of air gaps between the two glass plates. Loose particles, such as dust and glass debris, were removed by using the ultrasonic cleaner, whereas fused glass particles and burrs were efficiently removed by lapping the glass plates. An example of the laser micro-machined glass plate before and after the cleaning and lapping is shown in Fig. 4.

### Laser microwelding the glass plates

Our laser microwelding process uses a high-intensity, ultrashort pulsed laser beam, which is focused close to the interface of two solid materials of which at least one is transparent, to generate a plasma as a result of non-linear (multi-photon and/or tunnelling) absorption^56–58^. Thermal accumulation in the small volume around the plasma creates a melt region that after subsequent cooling locally fuses the two materials, creating a weld volume (called heat-affected zone, HAZ). Continuous weld seams are created by laterally translating the laser beam.

**Figure 5:**
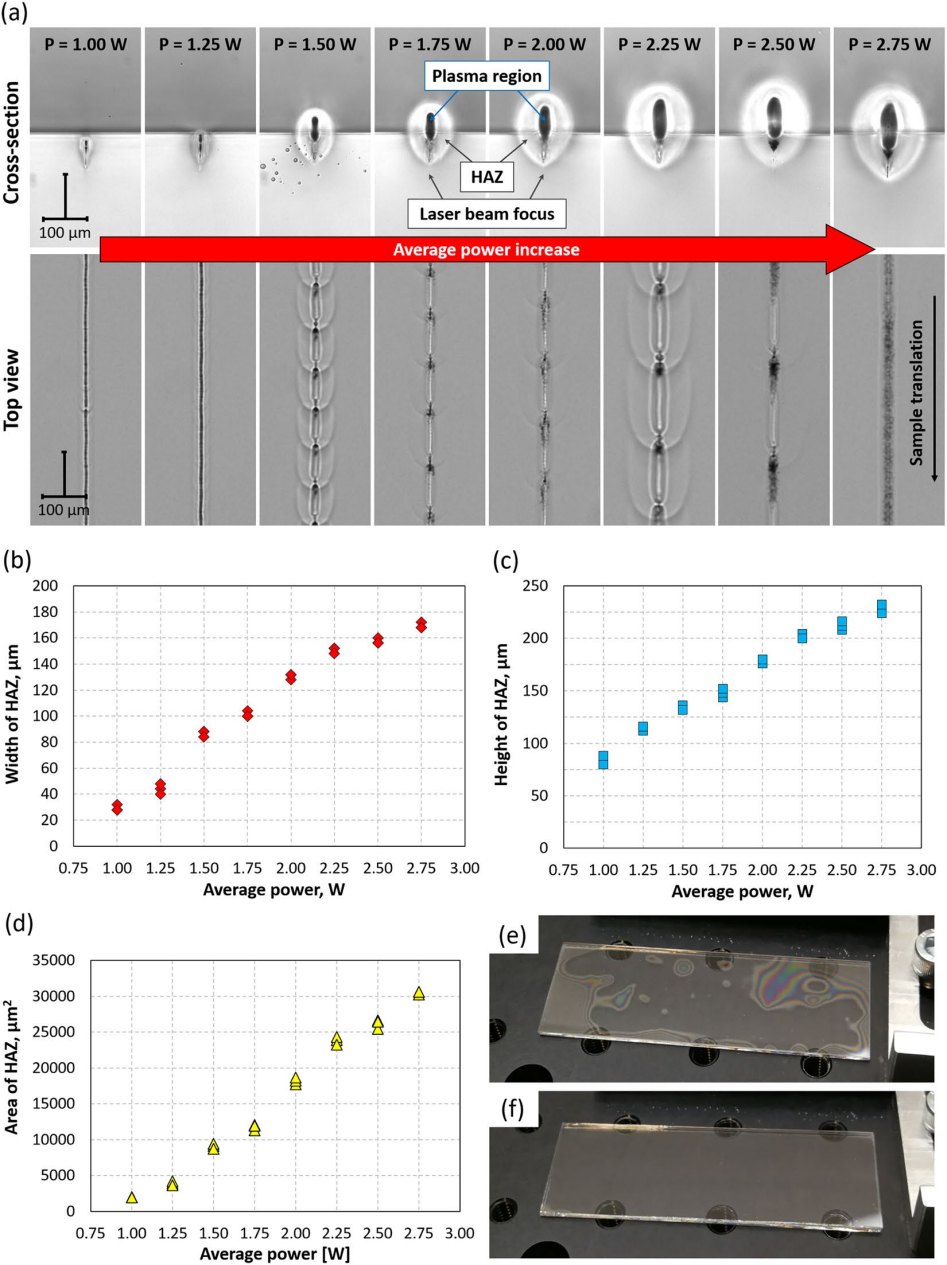
Laser welding results obtained with a sample translation velocity of 2 mm/s: (**a**) cross-section and top view of the weld seams generated at the glass-glass interface using different values of average power, (**b**) width, (**c**) height, and (**d**) area of HAZ from (**a**). Datasets used to plot these graphs are in the Supplementary Materials (see Table S2). A 0.5 mm thick glass plate on a 1.1 mm thick glass plate is shown in (**e**) after pre-bonding and (**f**) after welding, using P = 2.75 W and v = 2 mm/s. Weld seams in the form of parallel lines were generated at the glass-glass interface, covering the whole area of glass. The distance between the lines is 0.5 mm.

In order to generate weld seams inside glass, the ultrashort laser pulses must be delivered with a high PRF, of at least a few hundred kHz, to provide sufficient thermal accumulation^56^. In the case of Borofloat 33 glass, it was found that a PRF of 400 kHz is sufficient. The size of the HAZ and hence the cross-sectional area of the resultant welds depends on the average laser power (P) and the sample translation velocity (v). Figure 5(a) shows cross-sections and top views of the welds generated with a constant v of 2 mm/s and using different P values. The HAZ is clearly visible because the rapid resolidification of the melt results in a different refractive index from the surrounding material. These welds were generated by focusing the laser beam approximately 0.1 mm below the glass-glass interface. The top view of the weld seams shows that most of the welds have a periodic pattern and that this periodicity depends on the P value. This indicates that the laser-induced heat accumulated in the material undergoes regular terminations and the weld seams evolve continuously along with the laser beam movement^58^. For laser powers just above the process threshold (P = 1.00–1.25 W), the periodicity of the weld seams is less than a few tens of μm. These welds are very small, and hence are suitable for welding two very thin (≤250 μm thick) glass plates together. For laser powers between 1.5 and 2.5 W, the periodicity gets larger and the HAZ increases in size (see Fig. 5(b,c,d)). Interestingly, the periodicity was not observed in the weld seams generated at P = 2.75 W. At this laser power, the weld seams create a continuous line which seems to be optimal for bonding two glass plates together. Finally, it must be noted that the same periodicity patterns, as shown in Fig. 5(a), were also observed for the same laser power values when the laser beam focus was above or below the nominal position of 0.1 mm from the glass/glass interface (see Table S2 and Figures S1 to S8 in the Supplementary Materials).

In many cases the pre-bonding process (described in Materials and Methods) does not provide optical contact between the two glass plates over the entire area even though both glass plates are flat, smooth and relatively clean. Fortunately, the laser microwelding process itself reduces or even completely eliminates any existing air gaps between the two materials, as shown in Fig. 5(e,f). This is because the molten regions in both glass plates mix with each other and then shrink during cooling, pulling the two materials closer together. As shown in the Supplementary Materials (see Figure S9), welds can be generated exactly along the glass-glass interface in equally spaced locations.

### Replication of realistic rock samples

**Figure 6:**
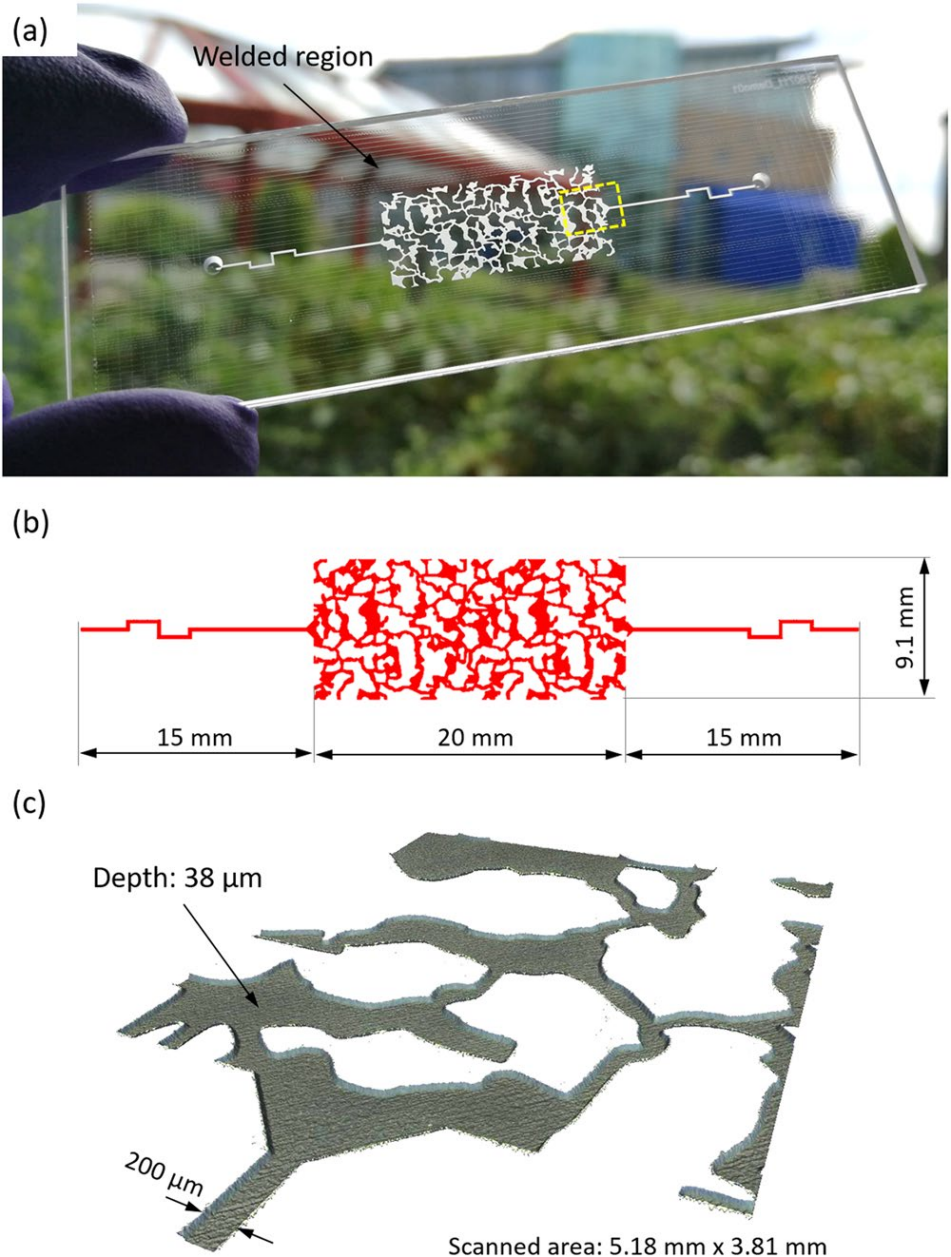
Microfluidic device containing irregular pattern that resembles an up-scaled 2D structure of Berea sandstone rock sample: (**a**) photograph, (**b**) pattern dimensions, and (**c**) 3D surface profile of the area selected in (**a**).

Figure 6 shows a microfluidic device in which the pattern resembles an up-scaled, thin section of a Berea sandstone rock sample^59^. The physical model was engineered at Schlumberger Cambridge Research (UK) and its thin section was measured using X-ray microtomography (XMT). This particular pattern has found use as a benchmark in many complex numerical simulations, such as direct numerical simulations using OpenFOAM software and lattice-Boltzmann simulations, and in pore-network modelling in order to simulate fluid flow and transport in complex 3D porous media^59,60^.

To generate the Berea rock pattern on a 1.1 mm thick glass substrate, firstly we saved the original image as a monochrome bitmap, then converted the bitmap into a DXF file (using an online converter available on: https://convertio.co/bmp-dxf/), and finally exported the DXF to TruTops PFO software. In the laser software, we selected areas for machining and defined laser process parameters, such E_P_, PRF, v, ΔH, number of passes, and laser beam raster scanning pattern. To make a longer flow pattern (20 mm × 9.1 mm), we tiled two patterns together.

The microfluidic pattern shown in Fig. 6 was generated using E_P_ = 42.8 μJ, PRF = 20 kHz, v = 40 mm/s, and ΔH = 6 μm. With two orthogonal laser passes (scan direction of ± 45°), we obtained the depth of 38 μm and the S_a_ value of approximately 1.2 μm. The whole pattern was generated in 18 minutes.

Inlet/outlet ports of 1.5 mm diameter were drilled in the second borosilicate plate (glass cover) using E_P_ = 62.8 μJ, PRF = 40 kHz, and v = 80 mm/s. To generate the through-holes, the glass cover was machined multiple times. After every 4 laser passes the glass plate was moved 0.15 mm towards the galvo scanner in order to maintain the laser beam focus on the machined surface. In each laser pass, the beam moved inwards the hole following a spiral pattern. For odd numbers of laser passes, the radius of the spiral decreased by 6 μm per revolution, whereas for even numbers of laser passes the radius of the spiral decreased by 5 μm per revolution. This laser beam scanning strategy allowed us to slightly reduce the machining time without affecting the surface finish of the holes.

Following the pre-bonding process, the glass plates were placed in the holder, as shown in Fig. 2(c), and pressed against each other by applying a constant pressure of 0.8 bar. Laser microwelding of the glass plates was performed using P = 2.75 W and v = 2 mm/s. The laser beam focus was 0.12 mm below the glass-glass interface. Welds were generated around the pattern and they can be seen as parallel and orthogonal lines in Fig. 6(a). The distance between these lines was typically 0.5 mm, but close to the microfluidic pattern the distance was in some cases reduced to 0.25 mm. The generated weld seams provide strong, permanent and watertight sealing of the patterns. Our tests have shown that the laser-generated weld seams can easily withstand injection pressures as high as 2.2 bar and temperatures up to 620 °C.

### Impact of the laser-generated surface texture on the flow of fluids

**Figure 7:**
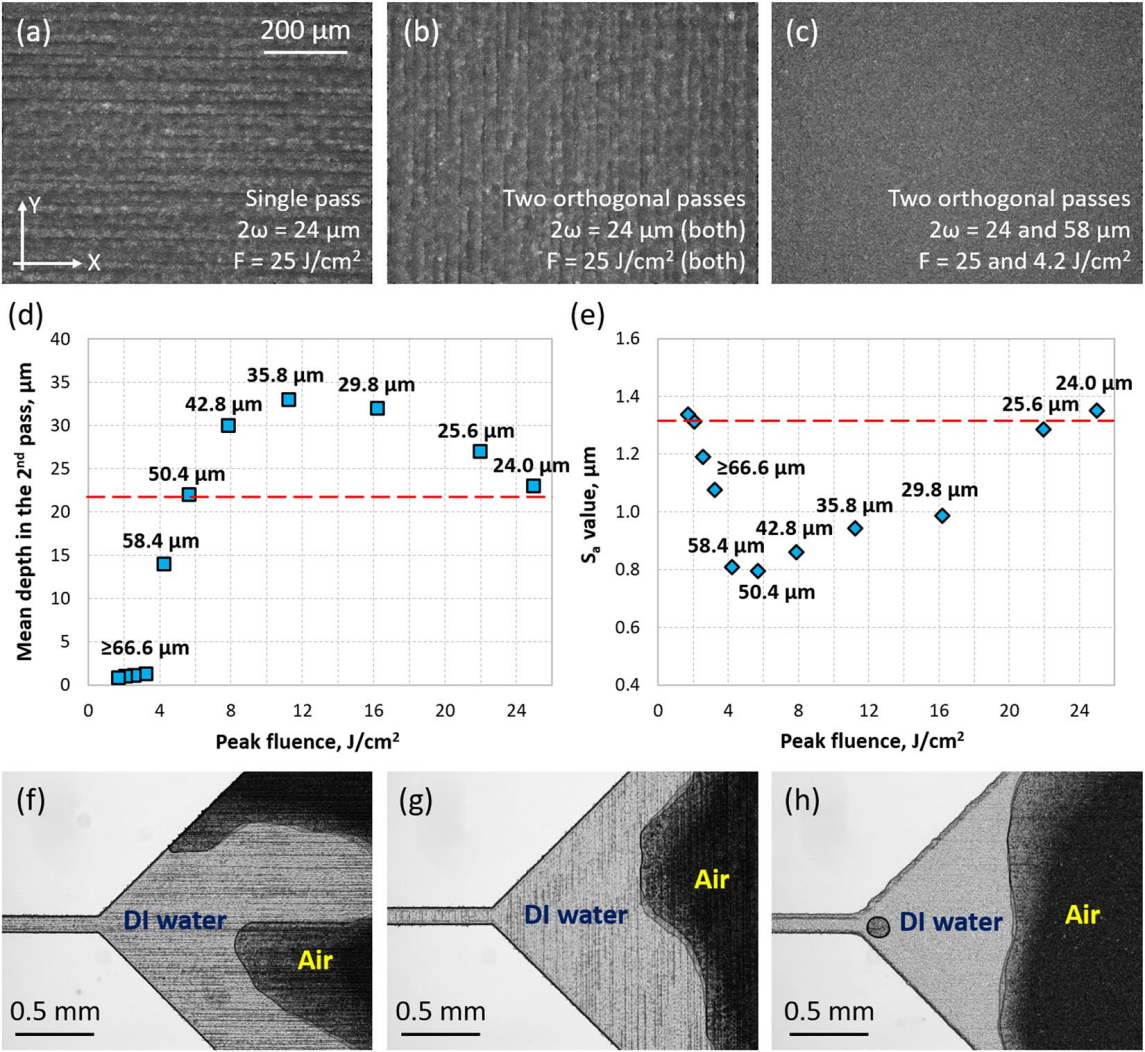
Glass surface after laser micromachining using: (**a**) single laser pass, laser spot diameter (2ω) of 24 μm, and peak fluence (F) of 25 J/cm^2^; (**b**) two laser passes orthogonal to each other using the same laser spot sizes and peak fluence values (2ω = 24 μm and F = 25 J/cm^2^); (**c**) two laser passes orthogonal to each other using two different laser spot sizes and peak fluence values (first pass: 2ω = 24 μm and F = 25 J/cm^2^, second pass: 2ω = 58 μm and F = 4.2 J/cm^2^). The other laser process parameters were as follows: PRF = 40 kHz, v = 80 mm/s, ΔH = 6 μm (bidirectional raster scanning). The relative mean depth and surface roughness (S_a_) after the second laser pass using 2ω ≥ 24 μm (see numbers above each data point), and hence for different peak fluence values, are shown in (**d**) and (**e**), respectively. The dashed lines show the mean depth and surface roughness obtained by the first laser pass using 2ω = 24 μm and F = 25 J/cm^2^. Finally, the results of spontaneous imbibition of deionised (DI) water by the surfaces (**a**), (**b**) and (**c**) are shown in (**f**), (**g**), and (**h**), respectively.

The surface of the laser-generated microfluidic patterns was observed to have a texture in the form of parallel ripples. These ripples are created as a result of the very small laser beam (typically 24 μm diameter) being translated across the material surface at high fluence. The orientation of these ripples was found to depend on the laser beam scanning direction in the final laser pass. Figure 7(a) shows a glass surface that was machined using 2ω = 24 μm and F = 25 J/cm^2^. The average roughness (S_a_) of this surface was measured to be 1.23 μm. As can be seen in Fig. 7(b), the next laser pass using the same laser parameters but a different laser beam scanning direction (perpendicular to the laser beam movement of the first pass) changes the orientation of the ripples and results in a small increase in surface roughness (S_a_ = 1.35 μm). Figure 7(c), meanwhile, demonstrates the reduction in surface roughness that can be achieved by using a larger laser beam in the second pass. By defocusing, the laser beam diameter (2ω) at the glass surface was increased from 24 to 58.4 μm (calculated using the Gaussian beam propagation equation), and this had the effect of completely removing the ripples and also reducing the S_a_ value to 0.8 μm (see Fig. 7(e)). In this case, melt phase was not observed, which implies that laser ablation is solely responsible for this smoothing effect. The second pass resulted in an increase in the mean depth from 22 to 36 μm. Additional results of this experiment can be found in the Supplementary Materials (see Table S3 and Figure S10).

The use of a laser spot diameter in the range of 24 to 50 μm also provides more efficient machining of Borofloat 33 glass in the second laser pass. As shown in Fig. 7(d), the mean depth can be even 50% greater than that obtained with the 24 μm diameter laser beam. The largest depth of 33 μm was obtained at the fluence of 11.2 J/cm^2^ where the 2ω was 35.8 μm. More efficient machining in the second pass results from an increased absorption of the laser beam, because the glass surface is rough and multiply scattering, and also from a higher laser beam overlap which results from a larger laser beam used for machining. Interestingly, the fluence of 11.2 J/cm^2^ is very similar in value to the ablation threshold determined for the first laser pass (see Fig. 3(a)).

Finally, it must be noted that the laser-machined glass surfaces are highly hydrophilic. The static contact angle between the glass surface and a droplet of deionised (DI) water was measured to be ≤ 20°. This means that a drop of DI water placed onto one of the inlet/outlet ports is imbibed spontaneously into the microfluidic pattern. During spontaneous imbibition of DI water, it was observed that the as-machined surface texture has a significant impact on the propagation of fluids. When the texture has ripples, see Fig. 7(f,g), DI water prefers to flow along these ripples, forming an “unrealistic” interface between DI water and air. Fortunately, the removal of these ripples by laser smoothing overcomes this problem, as shown in Fig. 7(h), and such surfaces can be used in the studies of subsurface systems.

### Other examples of glass microfluidic devices

**Figure 8:**
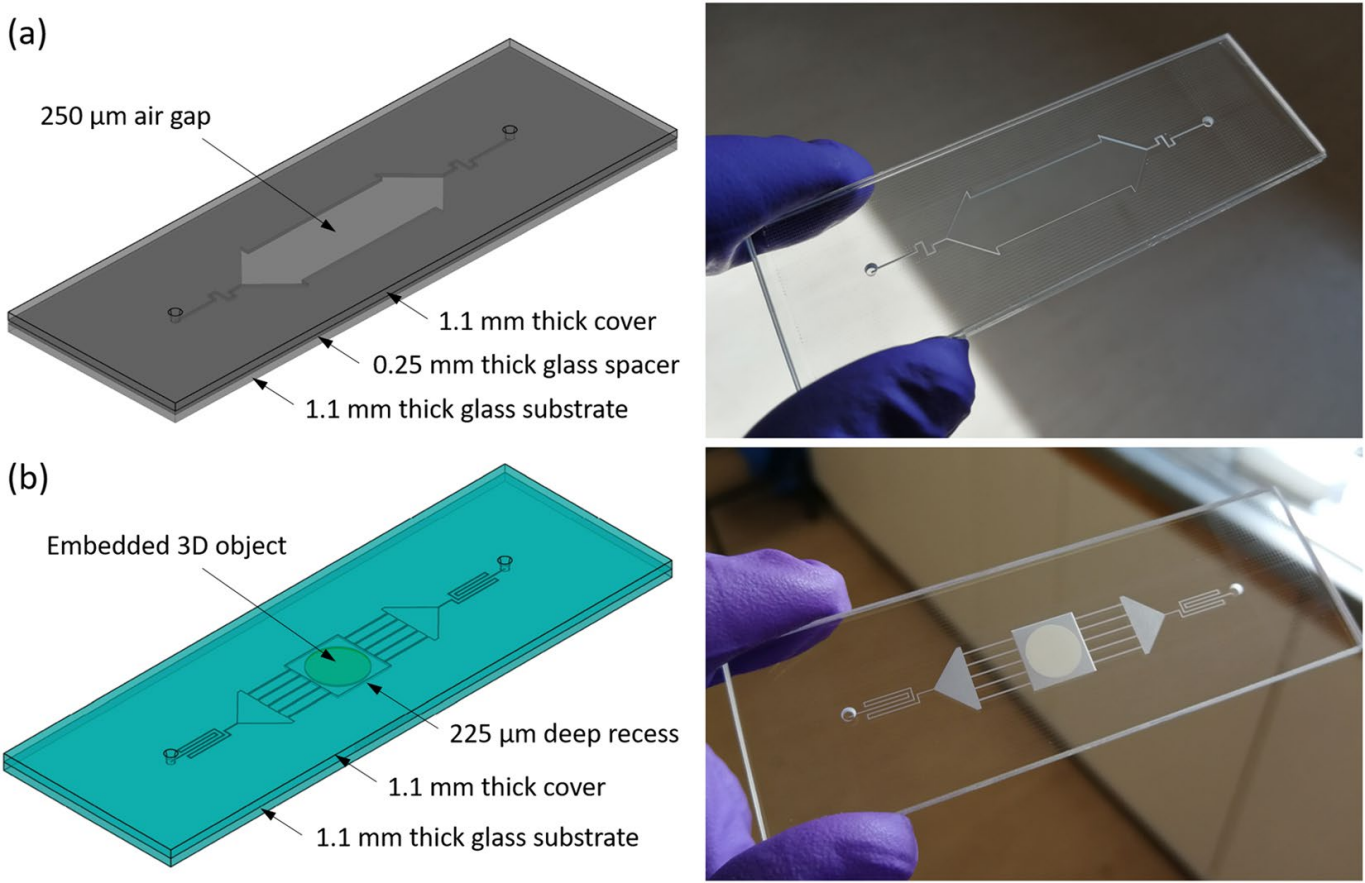
(**a**) Schematic drawing and photograph of a Hele-Shaw cell with a 0.25 mm air gap and (**b**) schematic drawing and photograph of a microfluidic device with embedded 3D object (a 175 μm thick piece of paper).

Our fabrication method also allows the manufacturing of Hele-Shaw cells, as shown in Fig. 8(a), which is fabricated from three glass plates. The thickness of the top and bottom glass plates was 1.1 mm, whereas the middle glass was only 0.25 mm thick. This thin glass plate was cut through using the picosecond laser (E_P_ = 62.8 μJ, PRF = 20 kHz, v = 40 mm/s, ΔH = 6 μm, 240 passes) in order to generate the microfluidic pattern. This plate is then used as a customised spacer between the two thicker glass plates, providing precise air gap between them.

Welding these three glass plates together was carried out in two steps. In the first step, the thin glass was welded to the bottom plate using P = 1.25 W and v = 2 mm/s. The laser beam was focused 75 μm below the glass-glass interface. For these laser welding parameters, the HAZ is relatively small (125 μm × 40 μm, as shown in Fig. 5(a)), which prevents the thin glass plate from cracking. Such a small power value also eliminates the risk of ablation of the upper glass surface. The second step was to weld the top glass to the thin glass plate. This time we used P = 2.85 W and v = 2 mm/s and the laser beam was focused 120 μm below the glass-glass interface.

Hele-Shaw cells of this type can be used for the investigation of various fluid flow mechanisms, including multiphase flow in homogenous media^52,61^. Moreover, if the air gap is filled with small glass beads this device can be used as a heterogeneous model of porous medium^62^. The pattern could be filled with glass beads just before the pre-bonding process or by injecting glass beads through one of the inlet ports.

The evidence that is possible to embed small 3D objects into glass microfluidic devices is shown in Fig. 8(b). Here, the microfluidic device comprises a 175 μm thick paper disc, which was inserted into a 225 μm deep square recess just before pre-bonding two glass plates together. The paper was used as a test piece in order to see whether the laser welding process may cause any damage to the objects sensitive to elevated temperatures, and as shown in Fig. 8(b) it remains intact. This means that small glass beads, thin sections of minerals or even small sensors can be integrated within glass microfluidic devices.

## Conclusions

In this report, we have described and demonstrated a maskless laser-based technique that is suitable for rapid prototyping and low volume manufacturing of different types of glass microfluidic devices. The technique is a flexible alternative to the time-consuming, multi-step conventional fabrication methods that typically involve using a combination of photolithography, etching and thermal/anodic bonding. Our technique uses a single tool, a picosecond pulsed laser system, to manufacture a fully-functional microfluidic device. The digitally-controlled micro-machining process directly generates microfluidic patterns that can be highly complex, allowing for instance an up-scaled replication of the cross-sections of realistic rock samples. By controlling laser machining parameters, it is possible to generate patterns with a variety of dimensions, aspect ratios, and surface morphologies.

The laser welding process, in turn, permanently bonds the two glass plates together without using any adhesives nor intermediate layers. The laser-generated welds are capable of closing sub-micron gaps between the two materials, and they are strong enough to withstand injection pressures of at least 2.2 bar (without using confined external pressure) and elevated temperatures as high as 620 °C. The laser-generated welds can be produced precisely at the glass-glass interface, which allows even very thin sheets of 250 μm thickness to be joined. The laser-generated microfluidic devices are fully watertight and can be used in many areas of research to investigate, for example, CO_2_ storage, water remediation, and hydrocarbon recovery processes.

Finally, our fabrication method enables the safe embedment of small 3D objects. This unique feature of the process opens an opportunity to manufacture glass microfluidic devices with integrated miniature sensors that can used to measure in real time different dynamic parameters (e.g. pressure, temperature or pH change) inside channels during the flow of fluids. By embedding such sensors into the patterns representing microstructures of realistic rock samples, it should be possible, for the first time, to obtain a better understanding of the fluid flow and reactive transport mechanisms that govern subsurface processes at pore level. These unique experimental data can be used, for instance, to validate the existing numerical models of subsurface systems, providing their further development and improvement of their accuracy.

## Supplementary information

Supplementary Information.

## Data Availability

All datasets used in this article can be found in the Supplementary Materials.
