# Supplementary material for: Maskless, rapid manufacturing of glass microfluidic devices using a picosecond pulsed laser
Source: Sci Rep. 2019 Dec 27;9:20215. doi: 10.1038/s41598-019-56711-5 (PMC6934552; doi:10.1038/s41598-019-56711-5)
Supplement: Supplementary file 1 — Supplementary Information. [file 41598_2019_56711_MOESM1_ESM.pdf]

Maskless, rapid manufacturing of glass microfluidic devices using a picosecond pulsed laser

Krystian L. Wlodarczyk[1,2,*], Duncan P. Hand[2], and M. Mercedes Maroto-Valer[1]

[1] *Research Centre for Carbon Solutions (RCCS), Institute of Mechanical, Process and Energy Engineering, School of Engineering and Physical Sciences, Heriot-Watt University, Edinburgh, UNITED KINGDOM*

[2] *Institute of Photonics and Quantum Sciences, School of Engineering and Physical Sciences, Heriot-Watt University, Edinburgh, UNITED KINGDOM*

* *Krystian L. Wlodarczyk; K.L.Wlodarczyk@hw.ac.uk; tel. +44 (0) 131 451 3105*

Contents of Supplementary Materials

Table S1. *Laser micromachining of Borofloat 33 glass by using λ = 515 nm. Results are given for a single laser pass and two laser passes. The datasets for a single laser pass were used to plot the graphs in Figure 3.* 3

Table S2. *Laser microwelding of 1.1 mm thick Borofloat 33 glass plates by using sample translation velocities (v) of 2, 4 and 6 mm/s. Datasets for v = 2 mm/s were used to plot the graphs shown in Figure 5. An area of the heat-affected zone (HAZ) was calculated by assuming that the cross-section of HAZ has an elliptical shape.* 4

Figure S1. *Cross-section and top view of the weld seams generated at the glass-glass interface by using laser powers (P) of 1.00 and 1.25W and a sample translation velocity (v) of 2 mm/s. The laser beam was focused at a distance Z (as indicated on images) below the top surface of the upper (1.1 mm thick) glass.* 6

Figure S2. *Cross-section and top view of the weld seams generated at the glass-glass interface by using laser powers (P) of 1.50 and 1.75W and a sample translation velocity (v) of 2 mm/s. The laser beam was focused at a distance Z (as indicated on images) below the top surface of the upper (1.1 mm thick) glass.* 7

Figure S3. *Cross-section and top view of the weld seams generated at the glass-glass interface by using laser powers (P) of 2.00 and 2.25W and a sample translation velocity (v) of 2 mm/s. The laser beam was focused at a distance Z (as indicated on images) below the top surface of the upper (1.1 mm thick) glass.* 8

Figure S4. *Cross-section and top view of the weld seams generated at the glass-glass interface by using laser powers (P) of 2.50 and 2.75W and a sample translation velocity (v) of 2 mm/s. The laser beam was focused at a distance Z (as indicated on images) below the top surface of the upper (1.1 mm thick) glass.* 9

Figure S5. *Cross-section and top view of the weld seams generated at the glass-glass interface by using laser powers (P) of 2.50 and 2.75W and a sample translation velocity (v) of 4 mm/s. The laser beam was focused at a distance Z (as indicated on images) below the top surface of the upper (1.1 mm thick) glass.* 10

Figure S6. *Cross-section and top view of the weld seams generated at the glass-glass interface by using laser powers (P) of 3.00 and 3.25W and a sample translation velocity (v) of 4 mm/s. The laser beam was focused at a distance Z (as indicated on images) below the top surface of the upper (1.1 mm thick) glass.* 11

Figure S7. *Cross-section and top view of the weld seams generated at the glass-glass interface by using laser powers (P) of 2.50 and 2.75W and a sample translation velocity (v) of 6 mm/s. The laser beam was focused at a distance Z (as indicated on images) below the top surface of the upper (1.1 mm thick) glass.* 12

Figure S8. *Cross-section and top view of the weld seams generated at the glass-glass interface by using laser powers (P) of 3.00 and 3.25W and a sample translation velocity (v) of 6 mm/s. The laser beam was focused at a distance Z (as indicated on images) below the top surface of the upper (1.1 mm thick) glass.* 13

Figure S9. *Cross-section of the weld seams generated at the interface of two glass plates. The laser power (P) used for welding was 2.5 W, whereas the sample translation velocity (v) was 2 mm/s. The laser beam was focused approximately 0.1 mm below the glass-glass interface. The distance between the welds is 0.5 mm. The images were taken using a Leica optical microscope with an objective zoom: (a) x5 and (b) x10. Please note that these images also show a welding line which was generated along the glass-glass interface using the same process parameters. For these laser welding parameters, the periodicity of the welds is observed.* 14

Table S3. *Laser machining of Borofloat 33 glass by using a defocused laser beam (see the 2ω values) at λ = 515 nm. These datasets were used to plot the graphs in Figure 7.* 15

Figure S10. *Glass surfaces after laser micromachining using: (a) a single laser pass, laser spot diameter (2ω) of 24 µm, and peak fluence (F) of 25 J/cm^2; (b) two laser passes orthogonal to each other using the same laser spot sizes and peak fluence values (2ω = 24 µm and F = 25 J/cm^2); (c)-(l) two laser passes orthogonal to each other using two different laser spot sizes and peak fluence values (first pass: 2ω = 24 µm and F = 25 J/cm^2, second pass: the 2ω and F values as listed in each image). The other laser process parameters were as follows: PRF = 40 kHz, v = 80 mm/s, ΔH = 6 µm (bidirectional sequential scanning). The first laser pass was always along the X axis, whereas the second pass was along the Y axis. The axes are indicated in Figure S9 (a).* 16

Table S1. Laser micromachining of Borofloat 33 glass by using λ = 515 nm. Results are given for a single laser pass and two laser passes. The datasets for a single laser pass were used to plot the graphs in Figure 3.

Single laser pass											
E_P [μJ]	2ω [μm]	F [J/cm²]	PRF [kHz]	v [mm/s]	ΔH [μm]	N [1/mm²]	E_{DOSE} [J/mm²]	Process time [s/mm²]	Depth [μm]	MRR [mm³/s]	S_a [μm]
24.6	24	10.9	20	40	2	250000	12.3	25.00	58.4	0.0023	1.37
33.6	24	14.8	20	40	2	250000	16.8	25.00	68.4	0.0027	1.65
42.8	24	18.9	20	40	2	250000	21.4	25.00	81.0	0.0032	1.79
52.2	24	23.1	20	40	2	250000	26.1	25.00	93.4	0.0037	1.91
61.7	24	27.3	20	40	2	250000	30.9	25.00	105.9	0.0042	2.06
71.1	24	31.4	20	40	2	250000	35.6	25.00	115.9	0.0046	2.20
24.6	24	10.9	20	40	4	125000	6.1	12.50	38.2	0.0031	1.10
33.6	24	14.8	20	40	4	125000	8.4	12.50	45.7	0.0037	1.23
42.8	24	18.9	20	40	4	125000	10.7	12.50	51.7	0.0041	1.37
52.2	24	23.1	20	40	4	125000	13.1	12.50	56.7	0.0045	1.52
61.7	24	27.3	20	40	4	125000	15.4	12.50	60.7	0.0049	1.51
71.1	24	31.4	20	40	4	125000	17.8	12.50	67.4	0.0054	1.60
24.6	24	10.9	20	40	6	83500	4.1	8.33	30.1	0.0036	1.03
33.6	24	14.8	20	40	6	83500	5.6	8.33	36.3	0.0044	1.04
42.8	24	18.9	20	40	6	83500	7.1	8.33	39.6	0.0048	1.05
52.2	24	23.1	20	40	6	83500	8.7	8.33	44.8	0.0054	1.07
61.7	24	27.3	20	40	6	83500	10.3	8.33	46.1	0.0055	1.00
71.1	24	31.4	20	40	6	83500	11.9	8.33	49.4	0.0059	1.01
Two laser passes											
E_P [μJ]	2ω [μm]	F [J/cm²]	PRF [kHz]	v [mm/s]	ΔH [μm]	N [1/mm²]	E_{DOSE} [J/mm²]	Process time [s/mm²]	Depth [μm]	MRR [mm³/s]	S_a [μm]
24.6	24	10.9	20	40	2	250000	6.1	12.5	27.1	0.0022	1.51
33.6	24	14.8	20	40	2	250000	8.4	12.5	35.4	0.0028	1.72
42.8	24	18.9	20	40	2	250000	10.7	12.5	40.1	0.0032	1.50
52.2	24	23.1	20	40	2	250000	13.1	12.5	47.3	0.0038	1.56
61.7	24	27.3	20	40	2	250000	15.4	12.5	51.6	0.0041	1.66
71.1	24	31.4	20	40	2	250000	17.8	12.5	57.3	0.0046	1.47
24.6	24	10.9	20	40	4	125000	3.1	6.25	19.4	0.0031	1.04
33.6	24	14.8	20	40	4	125000	4.2	6.25	21.9	0.0035	1.15
42.8	24	18.9	20	40	4	125000	5.3	6.25	25.0	0.0040	1.10
52.2	24	23.1	20	40	4	125000	6.5	6.25	27.6	0.0044	1.07
61.7	24	27.3	20	40	4	125000	7.7	6.25	29.9	0.0048	1.02
71.1	24	31.4	20	40	4	125000	8.9	6.25	31.9	0.0051	1.03
24.6	24	10.9	20	40	6	83500	2.1	4.17	15.5	0.0037	1.03
33.6	24	14.8	20	40	6	83500	2.8	4.17	18.6	0.0045	1.04
42.8	24	18.9	20	40	6	83500	3.6	4.17	19.7	0.0047	1.05
52.2	24	23.1	20	40	6	83500	4.4	4.17	21.6	0.0052	1.07
61.7	24	27.3	20	40	6	83500	5.2	4.17	22.9	0.0055	1.00
71.1	24	31.4	20	40	6	83500	5.9	4.17	24.7	0.0059	1.01

Table S2. Laser microwelding of 1.1 mm thick Borofloat 33 glass plates by using sample translation velocities (v) of 2, 4 and 6 mm/s. Datasets for v = 2 mm/s were used to plot the graphs shown in Figure 5. An area of the heat-affected zone (HAZ) was calculated by assuming that the cross-section of HAZ has an elliptical shape.

P [W]	v [mm/s]	Location of laser beam focus from the top surface of the upper glass [mm]	Width of HAZ [μm]	Height of HAZ [μm]	Area of HAZ [μm^2]	Mean periodicity of weld seams [μm]
1.00	2	1.15	28	88	1930	< 30
1.00	2	1.20	32	80	2010	< 30
1.00	2	1.25	28	88	1930	< 30
1.25	2	1.15	44	112	3870	< 50
1.25	2	1.20	48	112	4220	< 50
1.25	2	1.25	40	116	3640	< 50
1.50	2	1.15	88	132	9120	80
1.50	2	1.20	88	136	9400	90
1.50	2	1.25	84	132	8710	80
1.75	2	1.15	100	144	11300	100
1.75	2	1.20	104	148	12100	110
1.75	2	1.25	100	152	11900	100
2.00	2	1.15	128	176	17700	140
2.00	2	1.20	132	176	18200	140
2.00	2	1.25	132	180	18700	150
2.25	2	1.15	152	200	23900	150
2.25	2	1.20	152	204	24300	170
2.25	2	1.25	148	200	23200	170
2.50	2	1.15	156	208	25500	250
2.50	2	1.20	160	212	26600	220
2.50	2	1.25	156	216	26500	240
2.75	2	1.15	172	224	30300	> 750
2.75	2	1.20	168	232	30600	> 750
2.75	2	1.25	168	232	30600	> 750
2.50	4	1.15	168	220	29000	240
2.50	4	1.20	156	220	26900	310
2.50	4	1.25	144	216	24400	280
2.75	4	1.15	156	236	28900	> 750
2.75	4	1.20	152	232	27700	> 750
2.75	4	1.25	156	232	28400	> 750
3.00	4	1.15	168	228	30100	> 750
3.00	4	1.20	168	248	32700	> 750
3.00	4	1.25	168	244	32200	> 750
3.25	4	1.15	180	260	36800	> 750
3.25	4	1.20	172	276	37300	> 750
3.25	4	1.25	172	272	36700	> 750
2.50	6	1.15	136	216	23100	310
2.50	6	1.20	140	224	24600	380
2.50	6	1.25	132	224	23200	340
2.75	6	1.15	140	240	26400	> 750
2.75	6	1.20	140	240	26400	> 750

2.75	6	1.25	156	228	27900	> 750
3.00	6	1.15	148	236	27400	> 750
3.00	6	1.20	152	232	27700	> 750
3.00	6	1.25	160	232	29100	> 750
3.25	6	1.15	172	256	34600	> 750
3.25	6	1.20	172	248	33500	> 750
3.25	6	1.25	172	248	33500	> 750

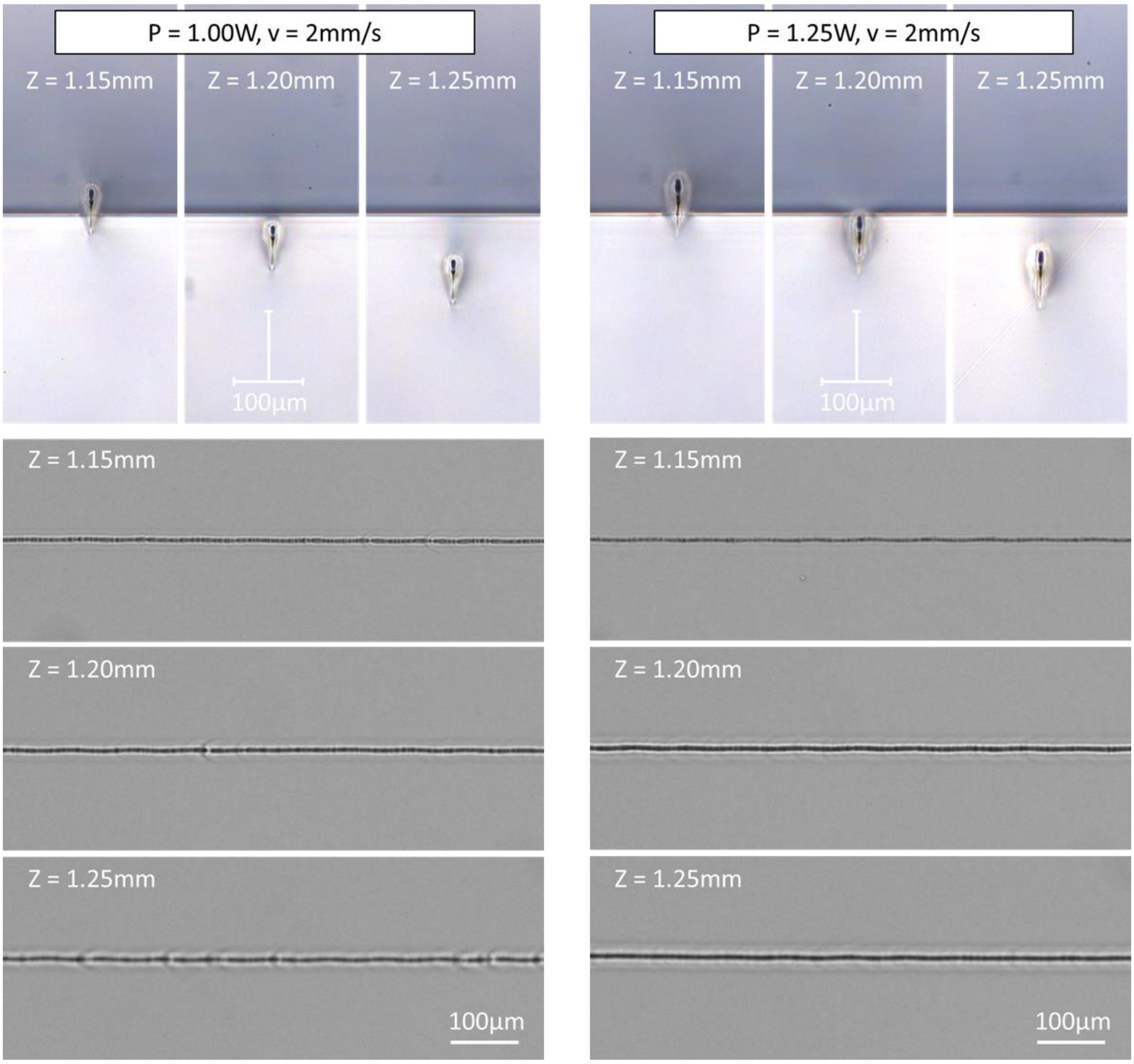

Figure S1. Cross-section and top view of the weld seams generated at the glass-glass interface by using laser powers (P) of 1.00 and 1.25W and a sample translation velocity (v) of 2 mm/s. The laser beam was focused at a distance Z (as indicated on images) below the top surface of the upper (1.1 mm thick) glass.

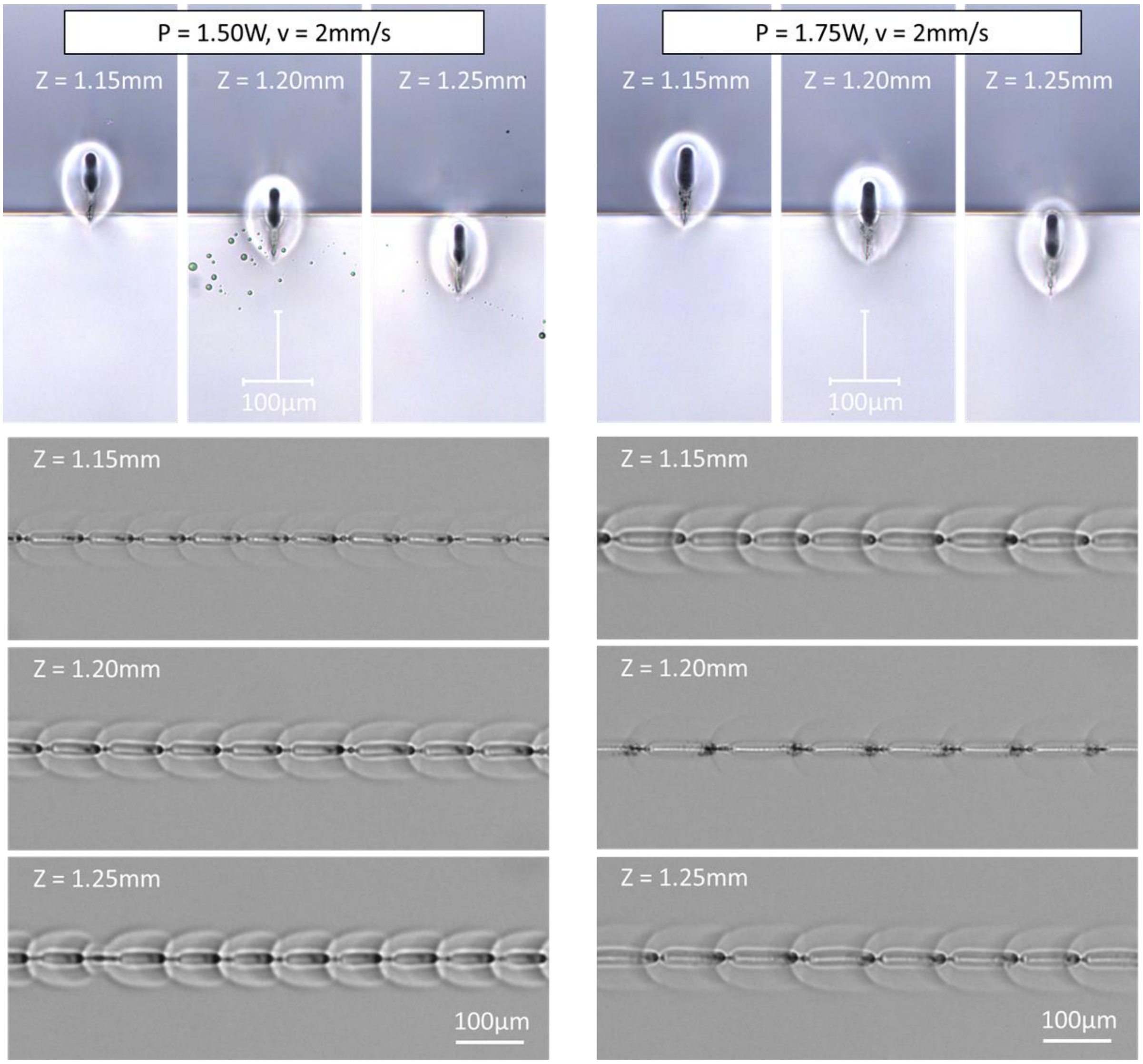

Figure S2. Cross-section and top view of the weld seams generated at the glass-glass interface by using laser powers (P) of 1.50 and 1.75W and a sample translation velocity (v) of 2 mm/s. The laser beam was focused at a distance Z (as indicated on images) below the top surface of the upper (1.1 mm thick) glass.

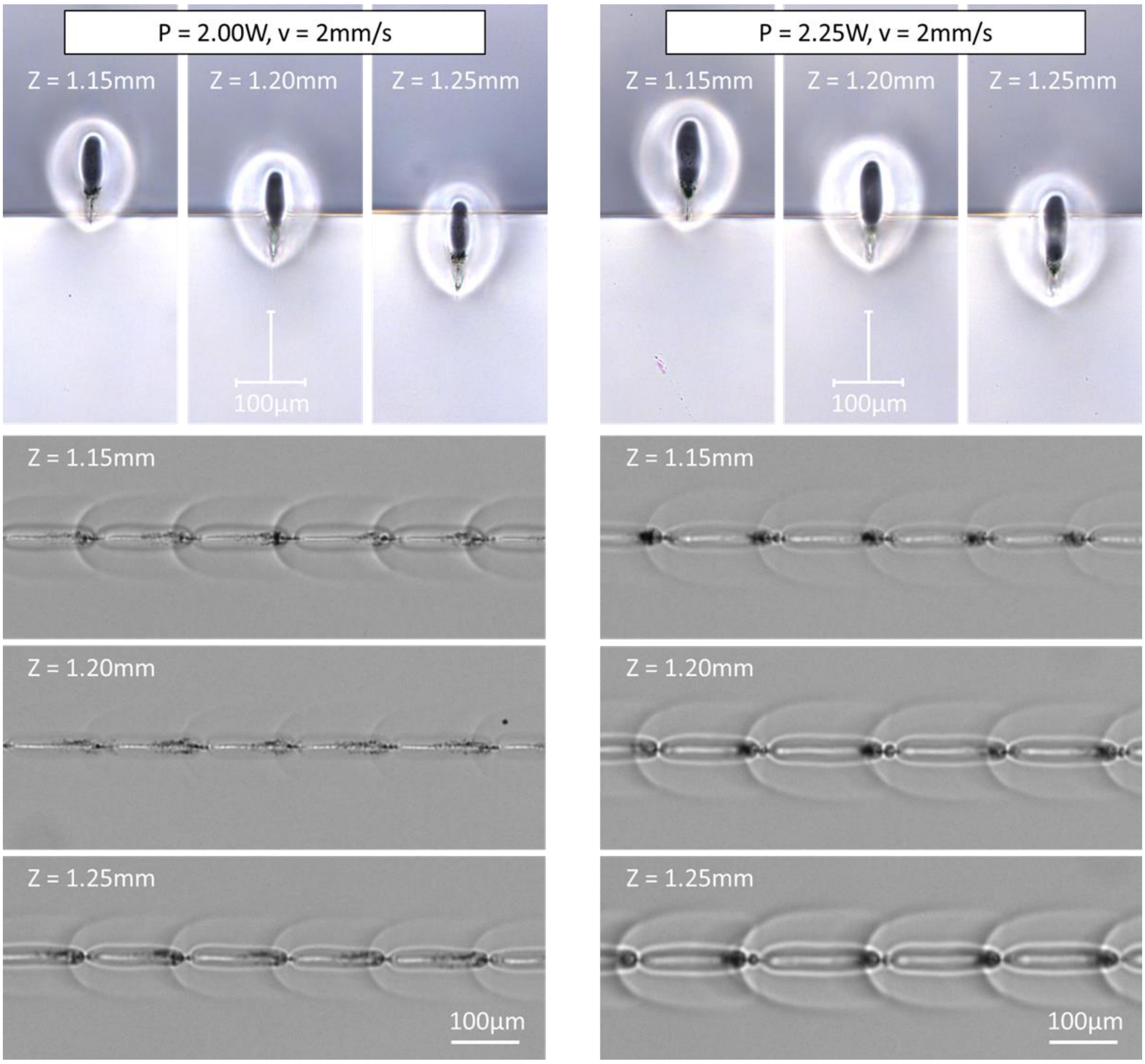

Figure S3. Cross-section and top view of the weld seams generated at the glass-glass interface by using laser powers (P) of 2.00 and 2.25W and a sample translation velocity (v) of 2 mm/s. The laser beam was focused at a distance Z (as indicated on images) below the top surface of the upper (1.1 mm thick) glass.

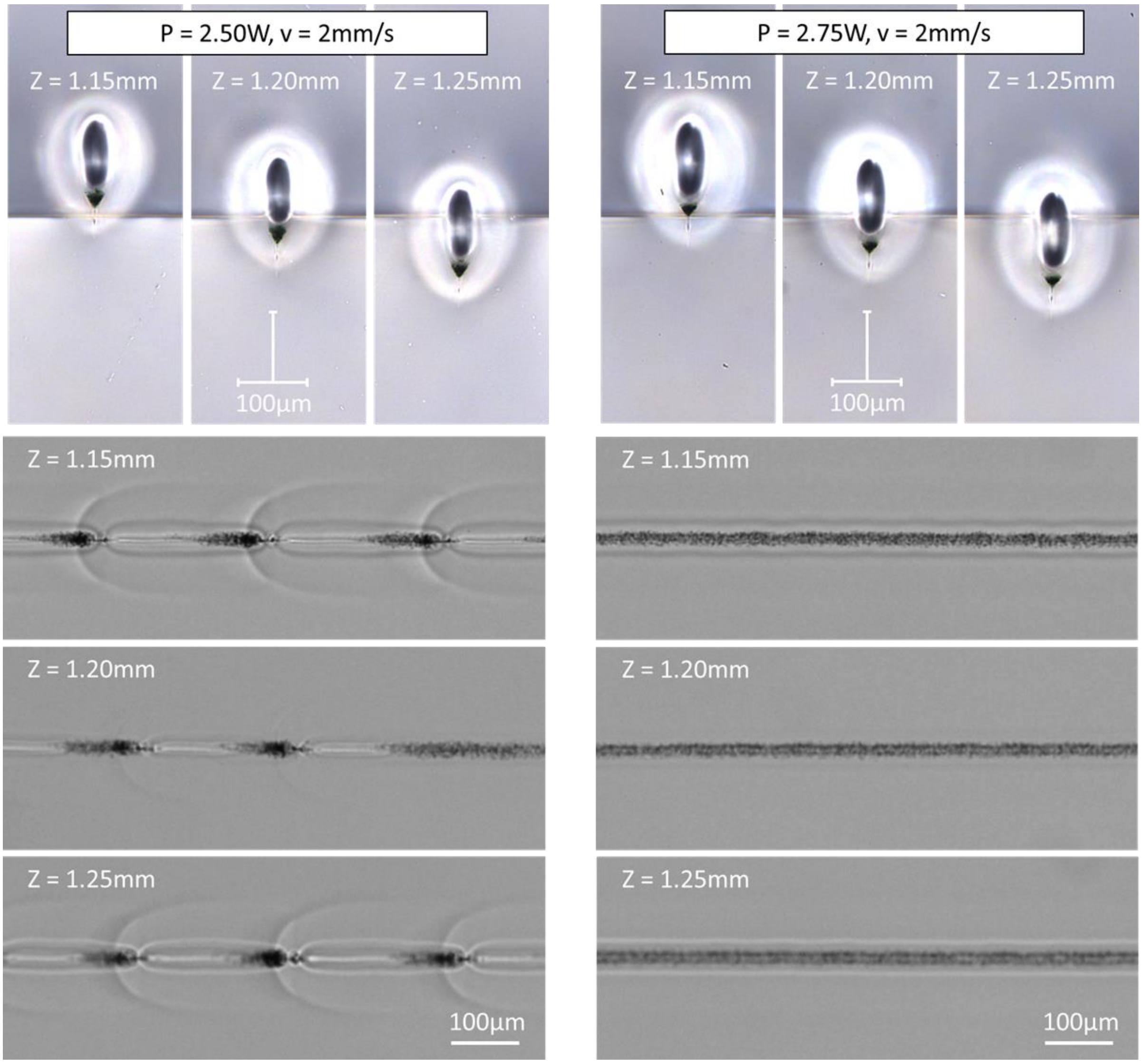

Figure S4. Cross-section and top view of the weld seams generated at the glass-glass interface by using laser powers (P) of 2.50 and 2.75W and a sample translation velocity (v) of 2 mm/s. The laser beam was focused at a distance Z (as indicated on images) below the top surface of the upper (1.1 mm thick) glass.

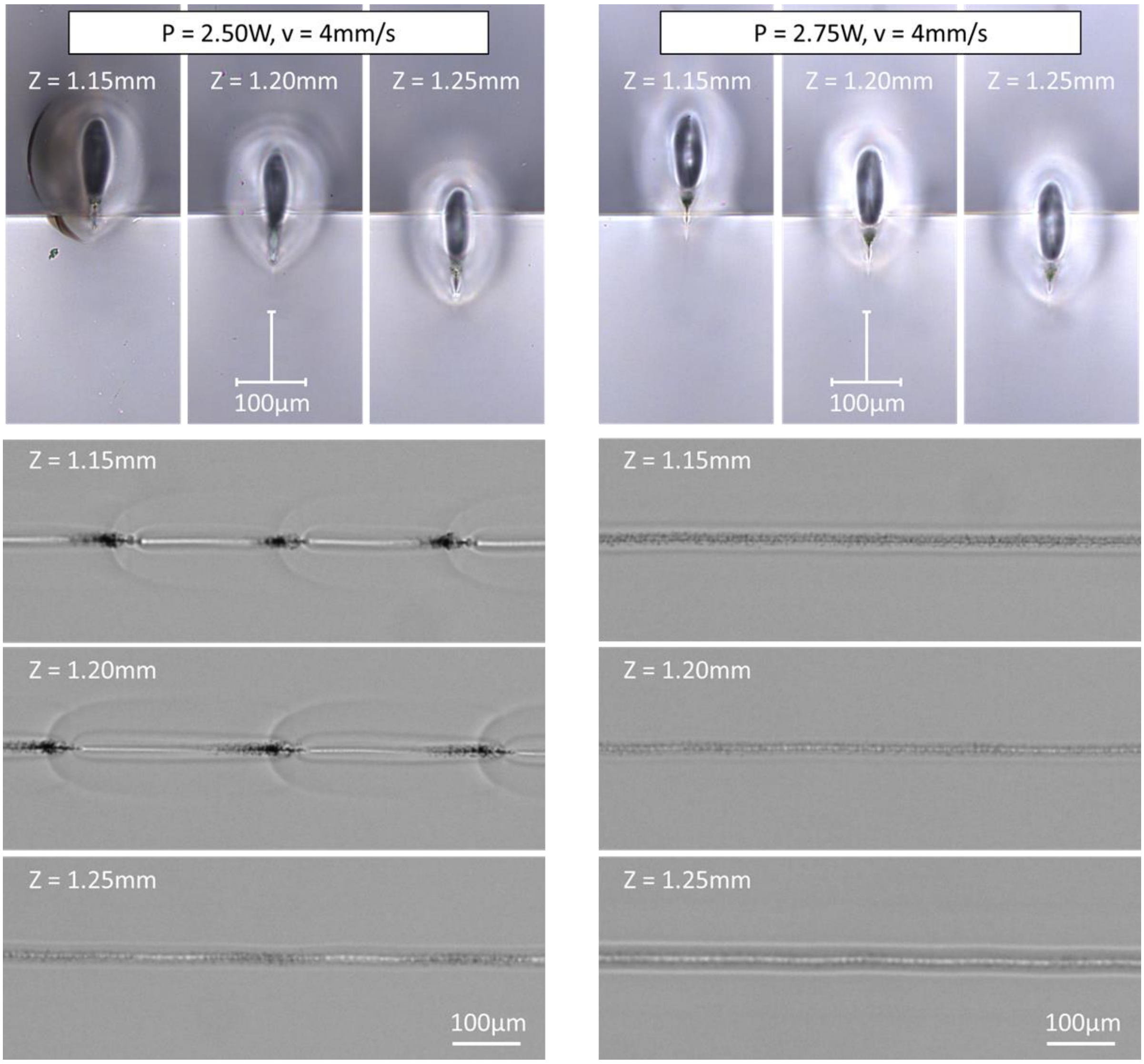

Figure S5. Cross-section and top view of the weld seams generated at the glass-glass interface by using laser powers (P) of 2.50 and 2.75W and a sample translation velocity (v) of 4 mm/s. The laser beam was focused at a distance Z (as indicated on images) below the top surface of the upper (1.1 mm thick) glass.

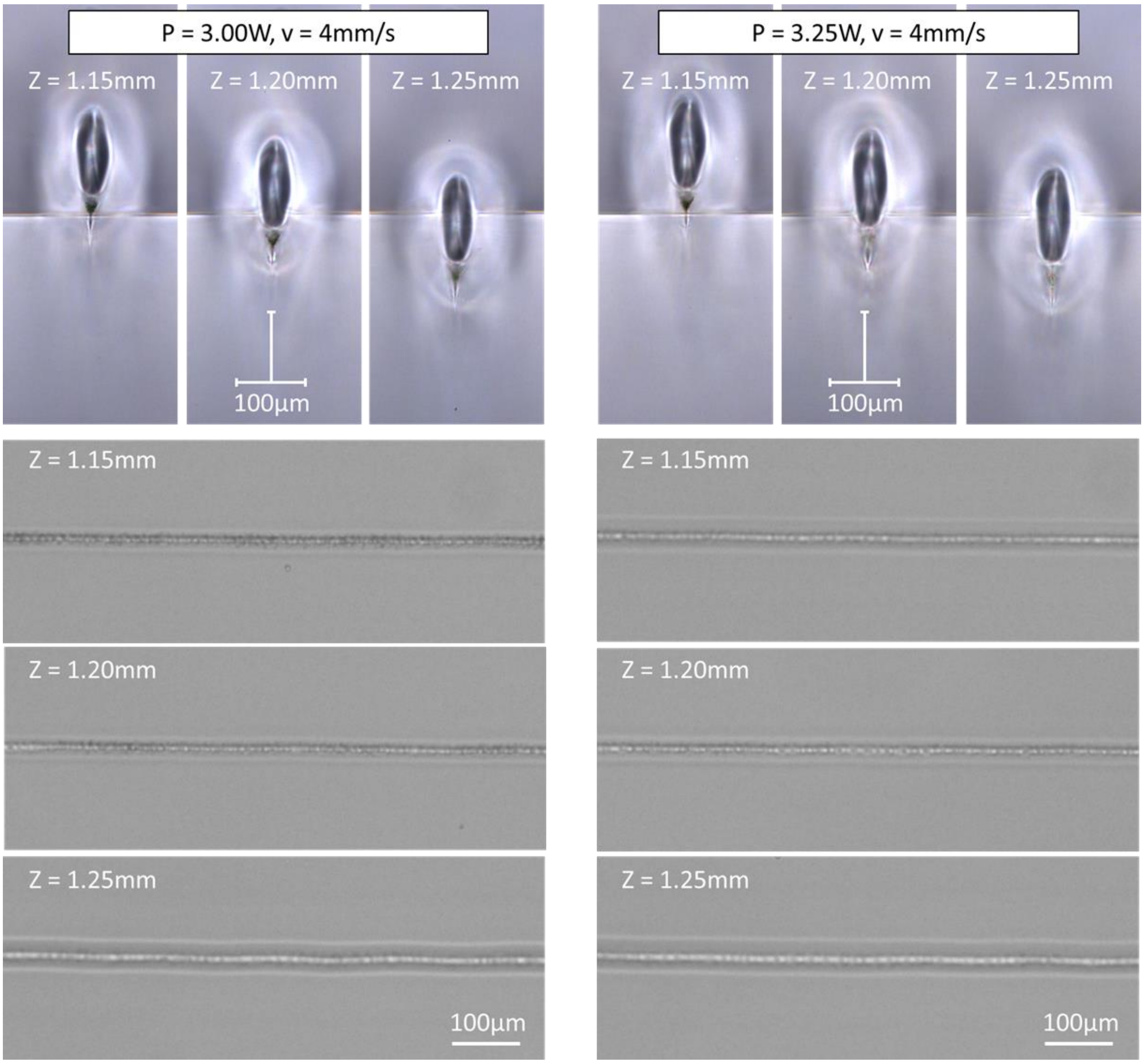

Figure S6. Cross-section and top view of the weld seams generated at the glass-glass interface by using laser powers (P) of 3.00 and 3.25W and a sample translation velocity (v) of 4 mm/s. The laser beam was focused at a distance Z (as indicated on images) below the top surface of the upper (1.1 mm thick) glass.

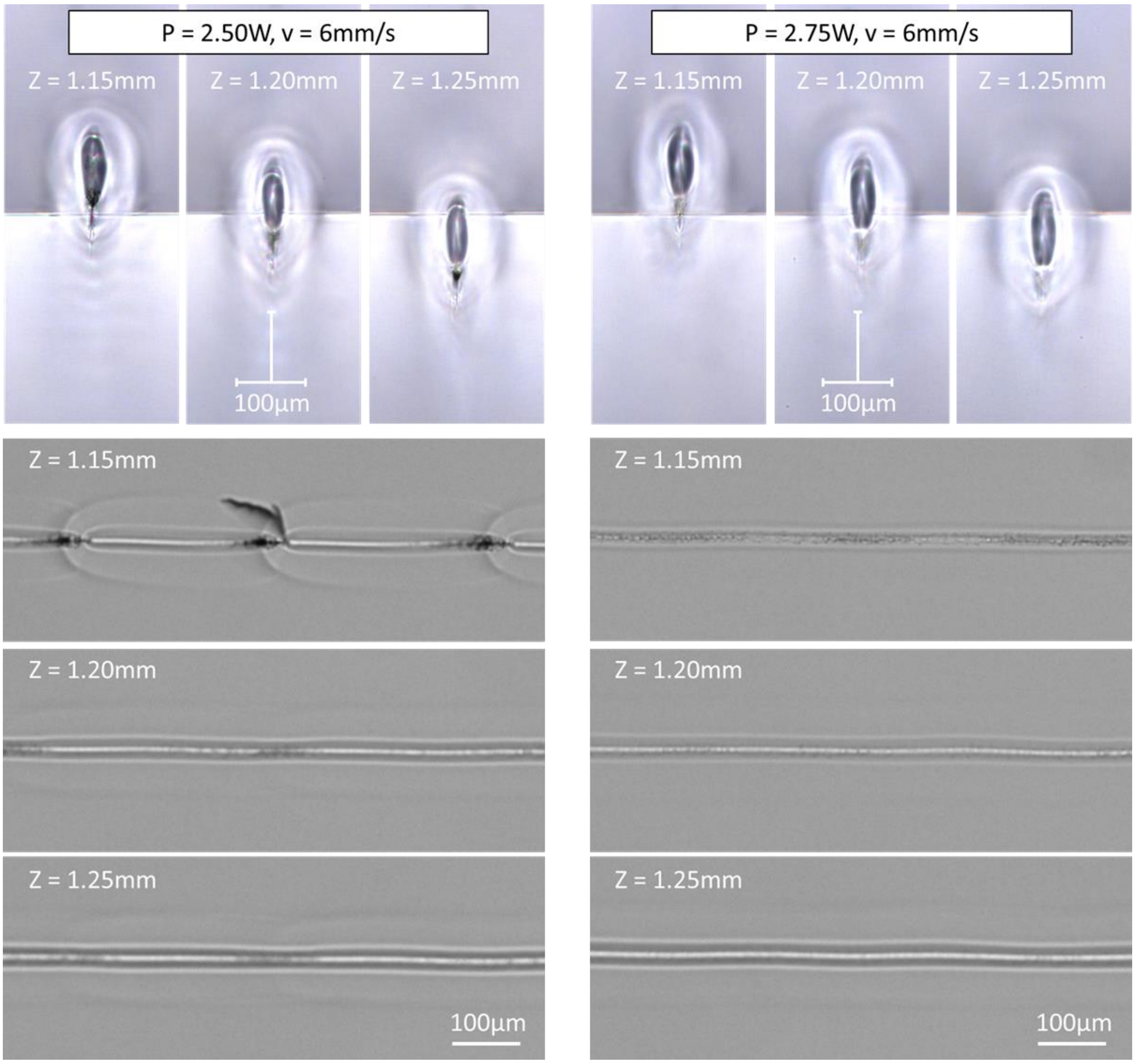

Figure S7. Cross-section and top view of the weld seams generated at the glass-glass interface by using laser powers (P) of 2.50 and 2.75W and a sample translation velocity (v) of 6 mm/s. The laser beam was focused at a distance Z (as indicated on images) below the top surface of the upper (1.1 mm thick) glass.

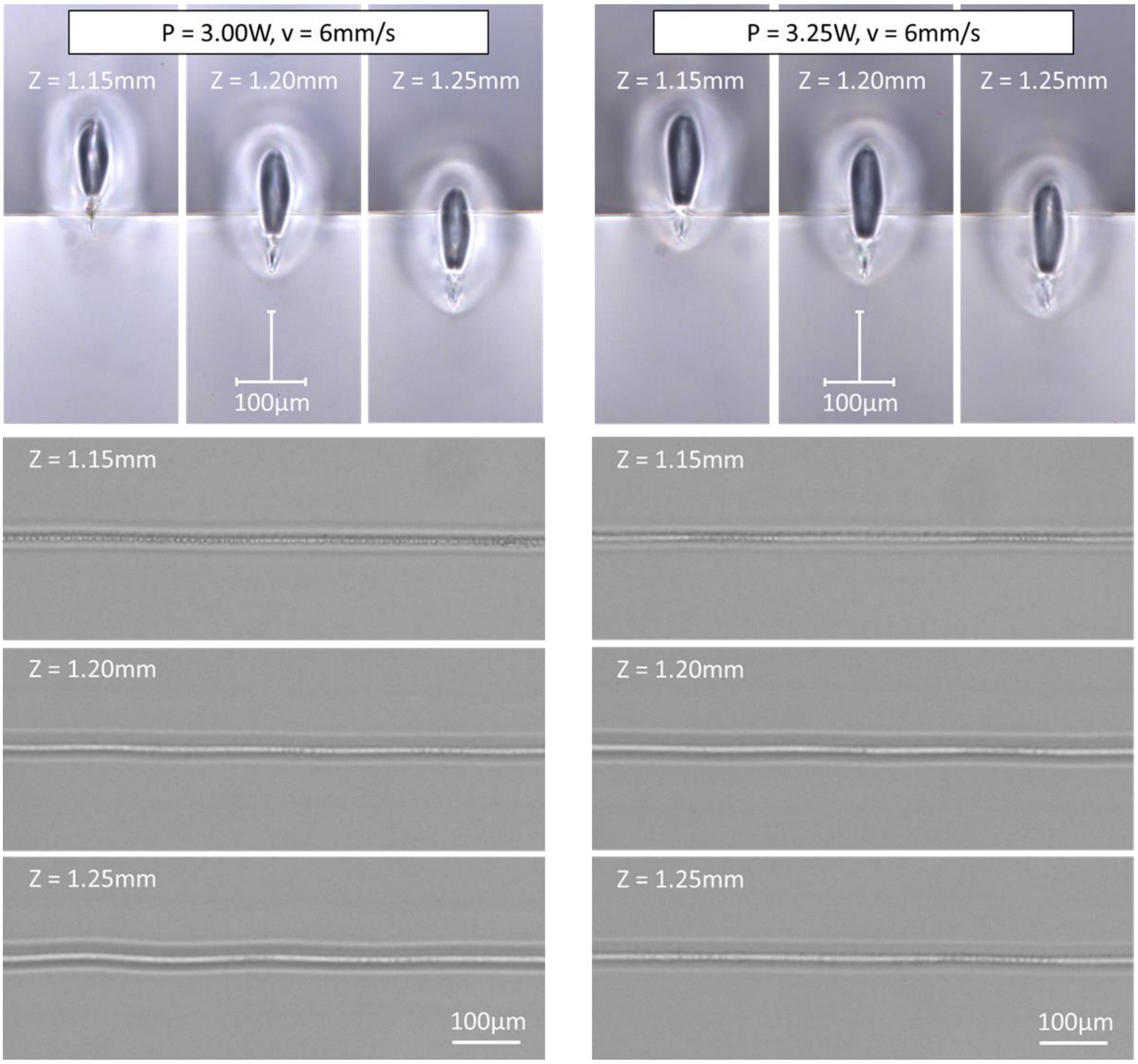

Figure S8. Cross-section and top view of the weld seams generated at the glass-glass interface by using laser powers (P) of 3.00 and 3.25W and a sample translation velocity (v) of 6 mm/s. The laser beam was focused at a distance Z (as indicated on images) below the top surface of the upper (1.1 mm thick) glass.

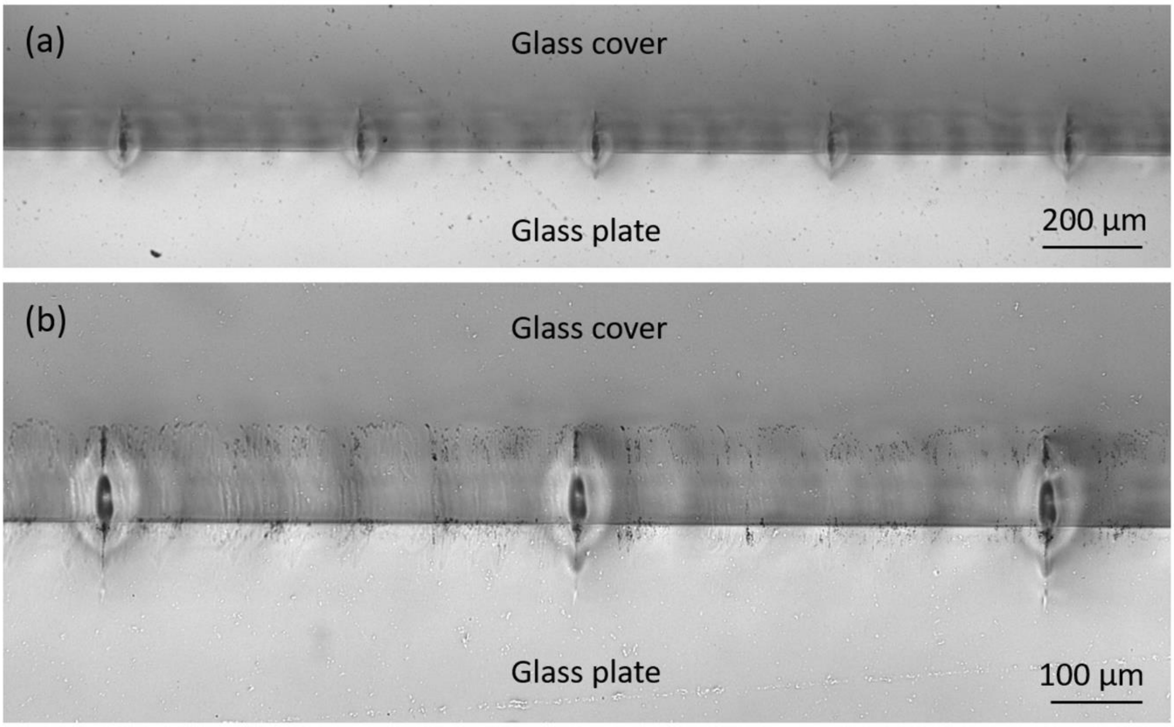

Figure S9. Cross-section of the weld seams generated at the interface of two glass plates. The laser power (P) used for welding was 2.5 W, whereas the sample translation velocity (v) was 2 mm/s. The laser beam was focused approximately 0.1 mm below the glass-glass interface. The distance between the welds is 0.5 mm. The images were taken using a Leica optical microscope with an objective zoom: (a) x5 and (b) x10. Please note that these images also show a welding line which was generated along the glass-glass interface using the same process parameters. For these laser welding parameters, the periodicity of the welds is observed.

Table S3. Laser machining of Borofloat 33 glass by using a defocused laser beam (see the 2ω values) at λ = 515 nm. These datasets were used to plot the graphs in Figure 7.

1st laser pass (horizontal)			2nd laser pass (vertical)			Absolute depth [µm]	Depth in 2nd pass [µm]	S_a [µm]
Z value [mm]	2ω [µm]	F [J/cm^2]	Z value [mm]	2ω [µm]	F [J/cm^2]			
0	24.0	25.0	-	-	-	22.0	0	1.23
0	24.0	25.0	0	24.0	25.0	45.0	23.0	1.35
0	24.0	25.0	0.25	25.6	22.0	49.0	27.0	1.29
0	24.0	25.0	0.50	29.8	16.2	54.0	32.0	0.99
0	24.0	25.0	0.75	35.8	11.2	55.0	33.0	0.94
0	24.0	25.0	1.00	42.8	7.9	52.0	30.0	0.86
0	24.0	25.0	1.25	50.4	5.7	44.0	22.0	0.79
0	24.0	25.0	1.50	58.4	4.2	36.0	14.0	0.81
0	24.0	25.0	1.75	66.6	3.2	23.3	1.3	1.08
0	24.0	25.0	2.00	75.0	2.6	23.1	1.1	1.19
0	24.0	25.0	2.25	83.4	2.1	23.0	1.0	1.31
0	24.0	25.0	2.50	92.0	1.7	22.8	0.8	1.34

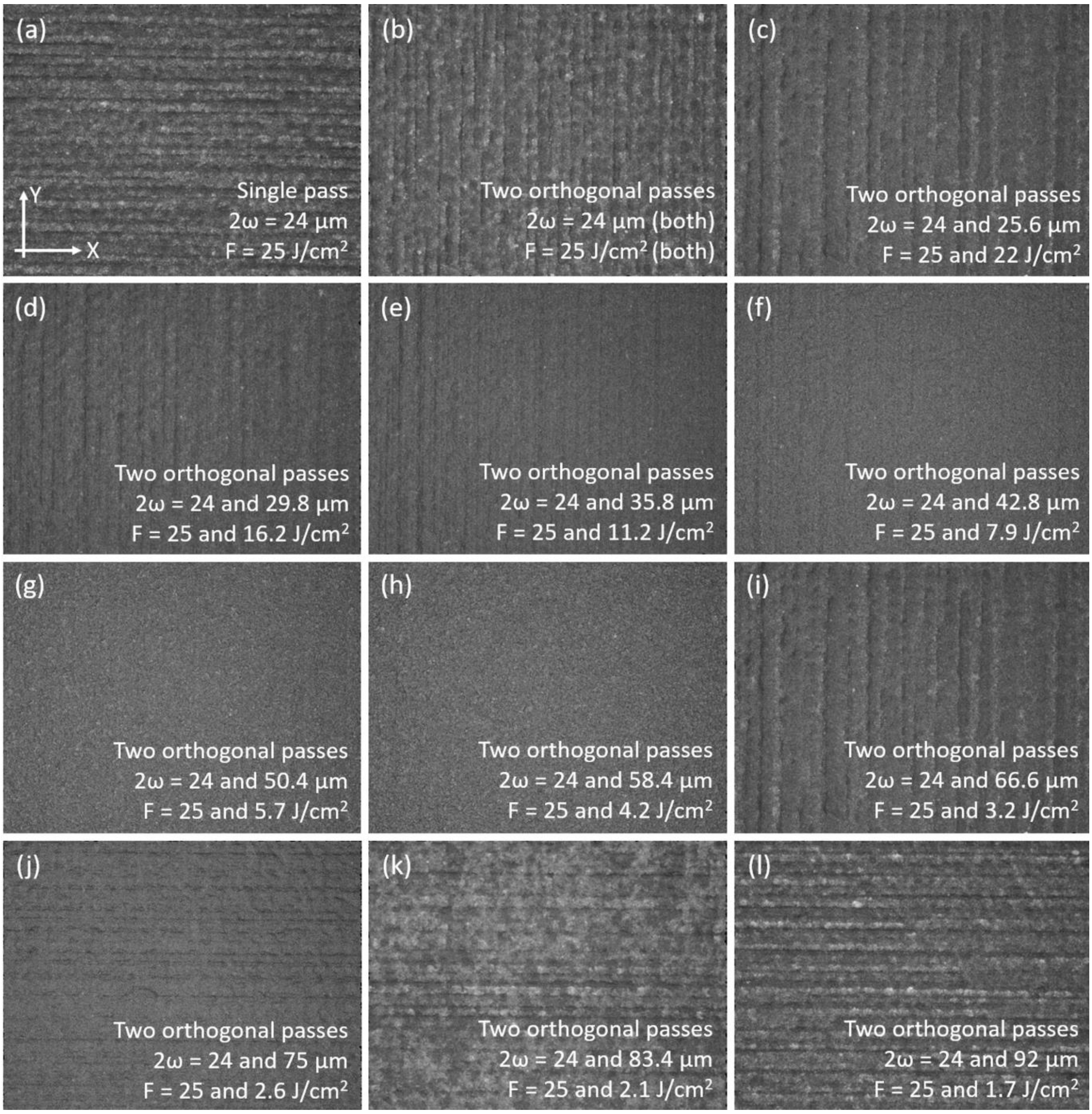

Figure S10. Glass surfaces after laser micromachining using: (a) a single laser pass, laser spot diameter (2ω) of 24 µm, and peak fluence (F) of 25 J/cm^2; (b) two laser passes orthogonal to each other using the same laser spot sizes and peak fluence values (2ω = 24 µm and F = 25 J/cm^2); (c)-(l) two laser passes orthogonal to each other using two different laser spot sizes and peak fluence values (first pass: 2ω = 24 µm and F = 25 J/cm^2, second pass: the 2ω and F values as listed in each image). The other laser process parameters were as follows: PRF = 40 kHz, v = 80 mm/s, ΔH = 6 µm (bidirectional sequential scanning). The first laser pass was always along the X axis, whereas the second pass was along the Y axis. The axes are indicated in Figure S9 (a).
